# Bodies of evidence: The human remains from Flinders Petrie’s excavations in British Mandate Palestine

**DOI:** 10.12688/openreseurope.18758.1

**Published:** 2025-01-23

**Authors:** Rachael Thyrza Sparks, Nina Maaranen

**Affiliations:** 1 University College London Institute of Archaeology, London, England, WC1H 0PY, UK; 2 Bournemouth University Department of Archaeology & Anthropology, Poole, England, BH12 5BB, UK

**Keywords:** archaeology, biodistance analysis, craniometrics, ethics of human remains, Karl Pearson, Flinders Petrie, Southern Levant

## Abstract

**Background:**

In the 1920s and 1930s Flinders Petrie excavated several sites in British Mandate Palestine (Tell Jemmeh, Tell Fara and Tell el-ʿAjjul), encountering numerous burials dating from the Chalcolithic period down to the Ottoman period. The osteological finds were thought to have been discarded, until the authors identified a curated selection of skeletal human remains from these tombs at the Duckworth Laboratory in Cambridge in 2017/2018.

**Methods:**

Rachael Sparks conducted archival research to explore how the human remains from Petrie’s excavations in the Southern Levant were recovered, recorded, curated and studied. This drew on original excavation records, contemporary publications, official and private correspondence, unpublished research notes, and the evidence of the human skeletal remains themselves.

Following on this archival investigation, Nina Maaranen conducted skeletal analyses on individuals from Bronze Age contexts – recording crania and mandibles using various non-invasive, macroscopic techniques to estimate age, sex and ancestry.

**Results:**

It was established that selected skulls were sent to Karl Pearson’s Biometric Laboratory at University College in London for craniometric study as part of wider programmes of research into ancient populations. After the war, changes in the organisation of the Eugenics Department at the University led to the transfer of Pearson’s collection of human skulls to the Duckworth Laboratory in Cambridge, where attempts to get the material published were unsuccessful.

The current skeletal analysis of the assemblage revealed a preference for adult individuals, in line with the curation motivations of the original investigators. Earlier research on these remains was compared with our new data and contextualised within the theoretical and methodological development of bioanthropology and osteology.

**Conclusions:**

Our investigation successfully identified the history of this assemblage, and revealed ethical issues surrounding the collection and subsequent use of some of these human remains, particularly where there may be familial links to modern Palestinian populations.

## Plain language summary

This article is the result of investigations into the archaeological and skeletal evidence from the excavations of Flinders Petrie in Palestine in the 1920s and 1930s, exploring contemporary attitudes to human remains, their treatment during and after excavation, and subsequent research history. The human remains from this assemblage were first transferred to the biometric laboratory at University College, then on to the Duckworth Laboratory in Cambridge, but despite several attempts to have these individuals studied, they remained unpublished and were subsequently forgotten by the academic community. Our rediscovery of these remains has allowed us to produce the first lab analysis since their excavation, connecting them to their archaeological contexts, while raising several ethical questions about Petrie’s motivations and methods.

## 1. Introduction

**Figure 1.**
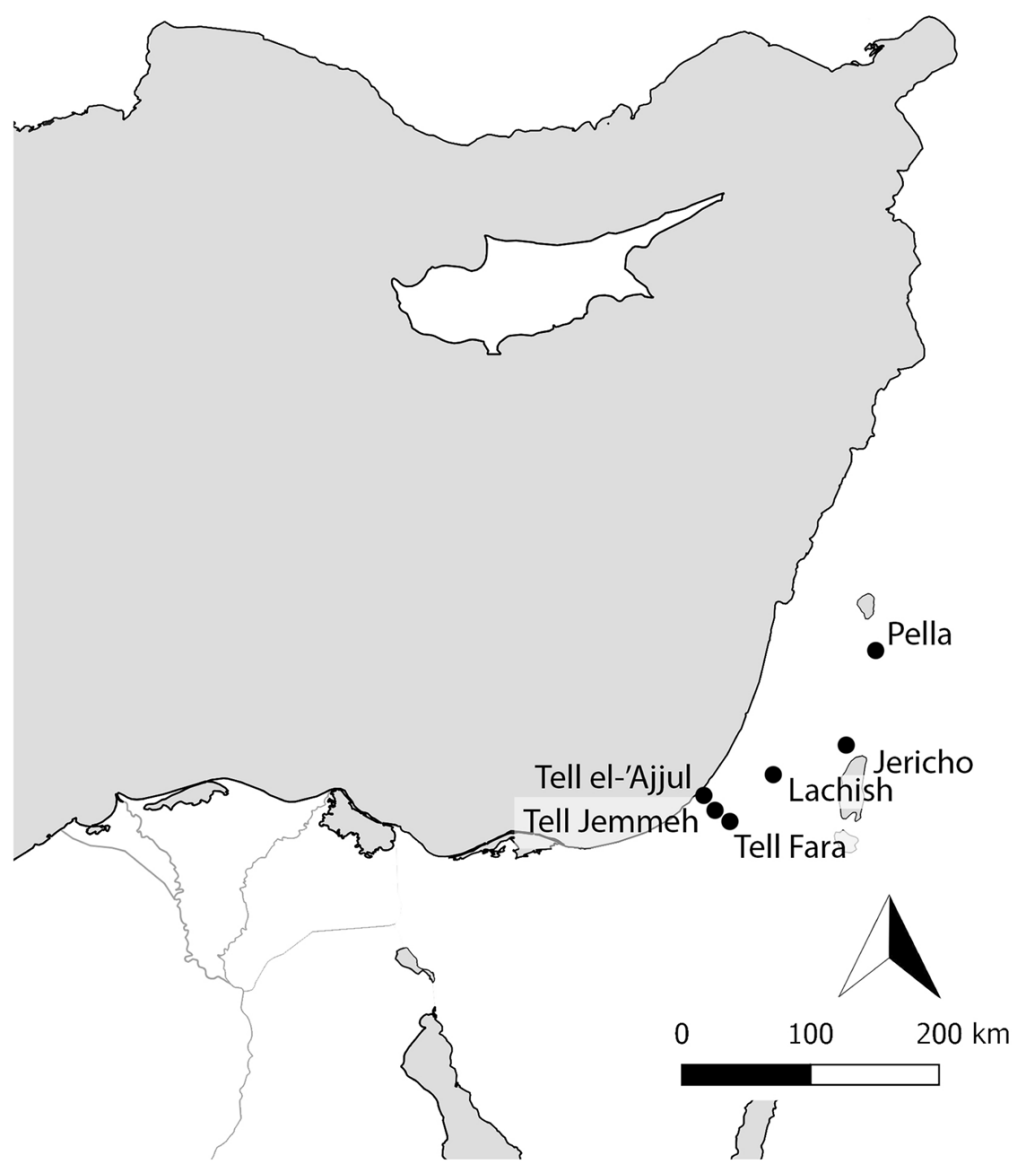
Map of sites discussed in the text. Illustration by Nina Maaranen.

The archaeologist William Matthew Flinders Petrie (1853–1942) worked extensively in Egypt before turning his attention to Southern Palestine in the 1920s. In both regions, he excavated numerous cemeteries, and following contemporary trends in osteological studies, collected large quantities of anthropometric data, as well as many physical skeletal human remains and skulls. The aim of this paper is to explore the way Petrie and his colleagues collected, curated, and investigated the human remains that they encountered, and to consider the ethical issues surrounding these assemblages and their potential for future research. Discussion will focus on evidence from the three main sites Petrie dug in British Mandate Palestine during the later part of his career: Tell Jemmeh, Tell Fara, and Tell el-ʿAjjul ( Figure 1).

The burial assemblages from Tell Fara and Tell el-ʿAjjul have been the subject of a number of detailed studies, both of whole cemeteries and specific burial groups ( Braunstein, 1998; Braunstein, 2011; Kennedy, 2015; Kenyon, 1956; Laemmel, 2003; Laemmel, 2009; Lehmann *et al.*, 2019; McClellan, 1979; Philips, 2023; Price-Williams, 1975; Price-Williams, 1977; Robertson, 1999; Sparks, 2013a; Tufnell, 1962; Tufnell, 1980a). Like most funerary assemblages, although these were recovered in a period where excavation methodologies and field recording left much to be desired from a modern standpoint, they still represent a series of important, largely-closed contexts that can provide invaluable insights into ancient funerary practice and ritual behaviours — explaining their continuing popularity with modern researchers. Common to all these studies is a focus on the material culture from the tombs rather than the human remains.

This was not due to any lack of interest on the part of the excavators, who recorded and published information about the bodies found, including orientation, position, sex and occasionally age. Nor was it due to poor state of preservation of the remains, as field records show a good level of detail was often available, while contemporary accounts describe their post-excavation care and storage, with particular attention paid to curation of the skulls, reflecting Petrie’s long-term interest in this type of material for anthropometric research ( Challis, 2016; Perry & Challis, 2013; Sheppard, 2010). And yet, while the information initially recorded has been used in later studies, especially the work of Braunstein (1998), there has been no direct study of the osteological remains themselves since the 1940s, when they largely vanished from public notice. Lack of information about where the material was stored even led some researchers to believe it had been discarded by the excavators (e.g., Braunstein, 1998: 35).

In 2017, Rachael Sparks located some of the human remains from Petrie’s Palestinian excavations in the Duckworth Collection in Cambridge; both authors subsequently made research visits to examine and record the collection in more detail. This paper is the result of these joint investigations. The aim is to reignite interest in this under-explored aspect of Petrie’s work by re-examining the human remains from Petrie’s excavations in Palestine and the circumstances under which they were assembled, curated, and brought to England, before being finally transferred to Cambridge. Evidence from field records, archives, and contemporary publications will be presented to show how Petrie excavated and recorded these burials. We will then consider his attitudes to the human remains he found, how they were treated, and what happened to this material after the close of excavations. Finally, we will present details of the extant osteological material known from these sites.

This paper aims to show that even the fragmentary evidence Petrie collected can still provide useful information, not only about past cultures, groups and individuals, but also about the early history of the discipline, and the intellectual framework in which early investigations were carried out. It is also hoped that this will direct specialists in the field to this important body of data, which is surely long overdue for re-examination and analysis.

## 2. Methods

The recovery and subsequent history of the human remains from these excavations was explored through a qualitative study of the archival records, with information has been drawn from a number of sources. These include contemporary publications relating to various excavations (e.g., Brunton, 1927; Macdonald *et al.*, 1932; Petrie, 1928a; Petrie, 1930a; Petrie, 1931a; Petrie, 1932a; Petrie, 1933b; Petrie, 1934a; Petrie *et al.*, 1952), and the methods employed ( Badè, 1934; Petrie, 1904; Petrie, 1934b). Documents relating to the running of the excavations may be found in the extensive archives of the British Mandate Department of Antiquities ( Israel Antiquities Authority, n.d.). The Olga Tufnell archive at the Palestine Exploration Fund is another important source, containing photographs and numerous letters written by Tufnell to her family while working on Petrie’s projects at Fara and ʿAjjul; some of her letters were recently published by Green and Henry (2021). Additional material has been drawn from the archives of the UCL Petrie Museum of Egyptology (relating to Petrie’s Egyptian work), the Petrie Palestine Archive at the UCL Institute of Archaeology (relating to his Palestinian excavations and including photographs, tomb cards and some field notebooks); and the Gerald Harding archive, also at the Institute of Archaeology, which includes photographs, short films, and a field diary from Tell Jemmeh ( Sparks, 2019).

Various documents relating to the deposition of skeletal human remains at collections in England, and its subsequent research were also consulted in the Karl Pearson Archive at UCL Special Collections, and in the archives of the Duckworth Laboratory, Museum of Archaeology and Anthropology at the University of Cambridge, Natural History Museum, and the British Museum. These contemporary accounts have also been supplemented with a range of autobiographical accounts, obituaries, and biographies and specialist studies (e.g., Challis, 2013; Clever, 2020; Crowfoot, 1943; Drower, 1995; Janssen, 1992; Murray, 1961; Parker, 1927; Petrie, 1930b; Pearson, 1938; Seton-Williams, 1988). Finally, there has been the evidence provided by the skeletal human remains themselves, and their associated field markings and documentation.

The osteological study was based on first-hand examination of the skeletal human remains. There was a focus on three aspects of skeletal biography; age, sex and biological distance (ancestry). Age-related skeletal changes are most commonly based on either developmental or degenerative processes, caused by intrinsic (e.g., genetic) and/or extrinsic (such as environmental) factors. Because the extant remains were mostly crania and mandibles, the macroscopic analysis of age was limited to few possible methods. For the few younger individuals, tooth development could be used to assess age, following the formation chart by AlQahtani *et al*. (2010). Once teeth have formed, the degree of cranial suture closure can be informative of broad age categories, i.e., young, middle and old adulthood. Sutures were assessed following Buikstra and Ubelaker (1994).

For the adult individuals (i.e., full permanent tooth development), sex was estimated by assessing the sexually dimorphic features of the skull; the Ascádi and Nemeskéri method, as presented in the standards by Buikstra and Ubelaker (1994), was used to assess the size and robusticity of glabella, supraorbital margin, nuchal crest, mastoid process and mental eminence. The mastoid process and the supra-orbital ridge have generally been reported most accurate in sex estimations ( Williams & Rogers, 2006), whereas the nuchal crest is the least accurate ( Garvin *et al.*, 2014).

Biological distance (henceforth biodistance) analysis conventionally uses skeletal and dental features, usually visible to the naked eye, as proxies for genetic data to estimate the biological closeness of individuals and groups. In archaeology, it has been particularly popular for estimating population histories and migration. Due to the preservation of the skulls, dental non-metric traits provided the best avenue of investigation; these traits are macroscopically visible features on the tooth crown and roots. Most dental non-metric traits are collected following the ASUDAS (Arizona State University Dental Anthropology System) standard. Studies comparing biodistance studies on ASUDAS traits with genetic studies have found positive correlations, particularly on the group level ( Delgado *et al.*, 2019). The data collection protocol has been outlined in other articles, e.g., Maaranen *et al.* (2022); the dental and oral traits were recorded following Scott and Irish (2017). Traits were not recorded in cases of moderate to strong dental attrition to avoid under- or over-scoring features. The side with the stronger expression was used and assumed to best express the underlying genotype.

## 3. Petrie’s excavations in Palestine

### 3.1 Tell Jemmeh

The first of our three sites to be excavated was Tell Jemmeh, located on the Wadi Ghazzeh (Nahal Besor), 10 km to the south of the modern city of Gaza ( van Beek, 1993: 667). It was dug in a single field season between December 1926 and May 1927. At the time, the site was identified as biblical Gerar ( Petrie, 1928a: 2), an identification which is no longer supported ( Van Beek, 1993: 667–668). Tell Jemmeh was occupied in the Chalcolithic period, then again from the Middle Bronze through to the Hellenistic period ( Van Beek, 1993: 668–673). The site was re-excavated between 1970 and 1990 by a team from the Smithsonian Institution, led by Gus Van Beek ( Ben-Shlomo & Van Beek, 2014).

Although Petrie’s team excavated several tombs during their work at Jemmeh, the site publication only mentions this material in passing ( Petrie, 1928a: 5, 12), with the most attention paid to the single Middle Bronze Age tomb found on a rise southwest of the cemetery ( Petrie, 1928a: 22, pl. LXII, top row). Several Roman tombs with limestone slab roofs were excavated somewhere off the tell, but said to be devoid of human remains ( Petrie, 1928a: 24), although this seems to be contradicted by the diary of one of his excavators, Gerald Harding, who reported around 20 bodies appearing in one of these tombs ( Sparks, 2019: 6–7). Medieval ‘Arab’ graves were also mentioned being present on the top of the tell, but were not described in any detail ( Petrie, 1928a: 25). Some of these produced multi-coloured glass bracelets, which might suggest a date somewhere from the Mamluk to Ottoman periods. It is worth noting that the Smithsonian excavations recovered a single burial in the uppermost phase at the site, which they could only date very generically somewhere between the Crusader/Mamluk period and the present ( Ben-Shlomo & Van Beek, 2014: 146).

There are no extant field records for any of these deposits, despite Petrie having a well-established system for recording burials that used pre-printed proformas known as Tomb Cards. The reason for this is not clear, as it would seem that Petrie directed his staff to investigate a possible cemetery area within a fortnight of beginning work at Jemmeh, suggesting that he was actively looking for graves ( Sparks, 2019: 7). It seems likely that any record keeping was done in field staff notebooks, which have not survived. Equally odd, though, is the lack of a system for numbering such tombs as were found. The Bronze Age tomb appears in the publication as ‘Cem. I,’ but no other tomb references appear to have been recorded.

### 3.2 Tell Fara

The second site to be excavated by Petrie was Tell Fara. This is located approximately 24 km south of modern-day Gaza, and is sometimes called Tell Farʿah South to distinguish it from a similarly-named site further to the north ( Yisraeli, 1993: 441). Tell Fara was excavated by Petrie and his field director Leslie Starkey over four field seasons, from 1927 to 1930; the final field season overlapped with work at nearby Tell el-ʿAjjul ( Petrie, 1930a; Macdonald *et al.*, 1932). Additional investigations were conducted at the site in 1976 ( Cohen, 1977), and then again between 1999 and 2002 ( Lehmann & Schneider, 2000; Lehmann, 2018; Lehmann *et al.*, 2018). The site was occupied from the Middle Bronze II period through to the Roman period ( Lehmann *et al.*, 2018; Yisraeli, 1993).

Over 500 ancient tombs were excavated at Tell Fara, located in a series of extramural cemeteries on the flat plain around the tell ( Lehmann *et al.*, 2019: Figure 2). The excavators assigned sets of tomb numbers to discrete cemetery areas, and these have become convenient names for their respective cemeteries: Cemeteries 100 (for Tombs 101–139), 200 (Tombs 201–272), 500 (Tombs 501–533), 600 (Tombs 601–663), 700 (Tombs 701–763), 800 (Tombs 801–864), 900 (Tombs 901–978) and 1000 (Tombs 1001–1027). Sadly, Petrie omitted the 1000 series tombs from his published plans, so the location of this cemetery is unknown. These cemeteries were in use from the Middle Bronze Age down to the Persian period, with only a handful of later, Roman tombs — essentially matching the periods of occupation on the tell itself.

Petrie did not officially report the presence of any intramural graves at Tell Fara, in contrast to Tell el-ʿAjjul (discussed below). Archival evidence suggests that this was a deliberate omission; numerous burials were actually cleared from the tell, and the skulls collected for future study. In a letter to anthropologist Ian Cunnison, Olga Tufnell described the background to this:

These skulls are modern, a tradition of burial on the mound was still very strong among the local Bedawin at Tell Fara, and some of the tribal marks found on the grave stones were still in use in 1927. However as these graves had to be ‘lost’ if excavation was to take place on the tell, they could not be referred to in any detail ( Tufnell, 1946c: 2).

Tufnell’s comments make it clear that these burials had grave markers and a likely connection to the existing population of the area. The presence of historically recent graves was subsequently confirmed by the excavations conducted by Ben-Gurion University, who discovered a later cemetery in a part of the tell not previously explored by Petrie (Area 2, Stratum 2–2). This cut into Roman levels, and had been disturbed by World War I military trenching ( Lehmann *et al.*, 2018: 118–119). Similar graves were also discovered in their Area 3 (Stratum 3–2); an Ottoman date was suggested for both ( Lehmann *et al.*, 2018: 119).

For a summary of the burial history of the site, see Table 1.

**Table 1.** Summary of the Tell Fara cemeteries and their periods of use.

Current name	Location	Tomb numbers	Periods of use
Cemetery 100	North of tell	101–139	LBIII, Iron I, Iron IIA, Iron IIB, Iron IIC, Hellenistic
Cemetery 200	Northwest of tell	201–272	Iron I, Iron IIA, Iron IIB, Iron IIC, Roman
Cemetery 500	Northwest of tell	501–533	MBII, MBIII, LBI, LBII, Iron I, Iron IIA
Cemetery 600	South of tell	601–663	MBII, MBIII, LBI, LBII, LBIII, Iron I, Persian, Hellenistic
Cemetery 700	West of tell	701–763	MBII, MBIII, LBI, LBIIA, Iron IIB, Iron IIC, Persian, Hellenistic
Cemetery 800	North of tell	801–865	LBIIB, LBIII, Iron I, Iron IIA, Iron IIB, Iron IIC, Persian, Hellenistic
Cemetery 900	Cut into the rampart on west side of tell	901–978	LBIIB, LBIII, Iron I
Cemetery 1000	Unknown	1001–1027	MBII, MBIII, Roman
Intramural	On top of the tell (Petrie excavations; Ben-Gurion excavations areas 2 and 3)		Ottoman

### 3.3 Tell el-ʿAjjul

The last of our three sites is Tell el-ʿAjjul, located in the territory of the Palestinian Authority, some 8 km southwest of Gaza ( Fischer, 2021: 141). Petrie excavated the site over five field seasons between 1930 and 1938 ( Petrie, 1931a; Petrie, 1932a; Petrie, 1933b; Petrie, 1934a; Petrie *et al.*, 1952). The final season was directed jointly by his colleagues Margaret Murray and Ernst Mackay, after Petrie was unable to secure an excavation license from the Palestinian Antiquities Department in his own name, due to disagreements in the quality of his field documentation ( Drower, 1995: 415–416). This did not prevent him working at the site as its unofficial director and presenting it as ‘his’ project to his supporters in England, causing some embarrassment to his colleagues who were forced to write to the Department disavowing Petrie’s comments ( Murray & Mackay, 1939).

New excavations were initiated at ʿAjjul by a Swedish-Palestinian mission in 1999, directed by Peter Fischer and Moain Sadeq, resulting in a variety of articles and short reports (e.g., Fischer, 2001a; Fischer, 2001b; Fischer, 2003; Fischer, 2004; Fischer, 2007; Fischer, 2009; Fischer, 2021; Fischer & Sadeq, 2000; Fischer & Sadeq, 2002). The project only lasted two field seasons before being prematurely ended by instability in the region; much of the site had since been destroyed by agricultural work, modern development and erosion ( Fischer, 2021: 143, 179). Consequently, much of the on-going research on ʿAjjul has continued to be based on re-evaluations of Petrie’s original work (e.g.: Bergoffen, 2001a; Bergoffen, 2001b; Kennedy, 2015; Kopetzky, 2011; Sparks, 2005; Sparks, 2013a; Sparks, 2021; Steel, 2002; Winter, 2018).

The earliest occupation at ʿAjjul appears to date to the Early Bronze IV period, when an area next to the tell was used as a burial ground ( Kennedy, 2015); at that time, the main settlement in the region appears to have been at nearby Tell es-Sakan ( Morhange *et al.*, 2005: 77). The tell itself was settled in the Middle Bronze Age, when the site became increasingly involved in maritime trade, especially with Cyprus ( Bergoffen, 2023; Sparks, 2021: 29). This may be the city recorded in Egyptian records as Sharuhen; if so, it may have been destroyed as part of the military campaigning of the pharaoh Ahmose, who is said to have besieged Sharuhen for three years before finally conquering it ( Kempinski, 1974: 149; Morris, 2005: 51–53; but cf Bergoffen, 2023). By the Late Bronze Age, ʿAjjul appears to have fallen under Egyptian control, with an Egyptian fortress constructed on the site ( Jeske, 2019: 191; Morris, 2005: 40–41). At this time, it became eclipsed by ancient Gaza, which gained status as a new centre of Egyptian administration for the region ( Katzenstein, 1982). However sporadic remains of the Iron Age, Hellenistic and Roman periods attest to continued activity at ‘Ajjul ( Tufnell & Kempinski, 1993: 52). There also appears to have been Islamic settlement on the uppermost level on the tell, attested by ceramic surface finds, pit contents and traces of architecture, which Petrie largely ignored ( Petrie, 1931a: 2, pl. XXXV.120; Petrie, 1932a: pl. XLVIII; Petrie, 1933b: 3, pl. LXIV).

As at Fara, Petrie focused a lot of attention on funerary remains, excavating over 1500 graves over the course of five field seasons. These included several extramural cemeteries, located on the flat plain around the northern and eastern sides of the tell. Sequences of tomb numbers were not allocated to discrete areas, as they had been at Fara, so Petrie adopted more generic names to describe the different cemetery areas; these are summarised in Table 2. The Lower Cemetery was located to the northwest of the mound ( Petrie, 1933b: pl. XLVIII; Petrie, 1934a: pl. LXIV). The Northeastern Cemetery was located east of the tell; Petrie named this the ‘18th Dynasty Cemetery,’ although it was actually in use throughout the whole of the Late Bronze Age and into the Iron II period, when it was used for a handful of cremations ( Culican, 1973: 68, n. 3; Petrie, 1932a: pls LI–LII). South of this is another cemetery, divided by an east–west tunnel, and excavated in the first field season; Petrie called the area north of the tunnel the ‘Hyksos Cemetery’ and that south of the tunnel the ‘Copper Age Cemetery,’ but the whole area is now referred to in the literature as the Eastern Cemetery ( Petrie, 1931a: pl. LV; Petrie, 1932a: pl. LI). It is not to be confused with an additional extramural cemetery, excavated in the second field season, which Petrie also called the ‘Copper Age Cemetery’. This has since been re-dated to the EBIV period, and is now usually referred to as the 1500 Cemetery, as it contained most of the 1500-series tombs ( Kenyon, 1956; Petrie, 1932a: 2, pl. LI). Usage of several of these extramural cemeteries overlapped in time ( Gonen, 1992: 70–82). A few additional tombs had been cut into the sides of the fosse around the eastern side of the tell ( Petrie, 1932a: pl. LII).

**Table 2.** Summary of the Tell el-ʿAjjul cemeteries and their periods of use. EB = Early Bronze Age, MB = Middle Bronze Age, LB = Late Bronze Age.

Current name	Petrie’s terminology	Location	Tomb series represented	Periods of use
Lower Cemetery	Lower Cemetery	Extramural, north of the tell	300, some original 1500-series, 1600, 1800, 1900; some duplicate 400-series tombs.	MBIII, LBI, LBII, LBIII
Northeastern Cemetery	18th-Dynasty Cemetery	Extramural, northeast of the tell	800, 1000, 1100	MBIII, LBI, LBII, Iron I, Iron II
Eastern Cemetery	Hyksos Cemetery (area north of the tunnel), Copper-Age Cemetery (area south of tunnel)	Extramural, east of the tell	100, 200, original 400-series	EBIV, MBIII, LBI, LBIIA
1500 cemetery	Copper-Age Cemetery	Extramural, 0.25 miles west of the tell	Some original 1500 series	EBIV, MBIII? ^1^
Fosse	Fosse	Extramural, east side of the tell	1100	MBIII, LBI, LBII
Courtyard Cemetery	Courtyard Cemetery	Intramural, north end of site	1400	MBI, MBII, MBIII, LBII? ^2^
Intramural burials		Across site: areas A, B, D, E, F, G, J, LA, LZ, T	500, 600, 1200, 1300, some 1400, 1700, some 1900, 2000, 2100; some duplicate 400-series and duplicate 1500-series tombs	MBII, MBIII, LBI, LBIIA

Numerous ancient intramural burials were also found up on the tell, often dug into open or abandoned parts of the settlement ( Petrie, 1931a: pl. LIV; Petrie, 1932a: pls XLVI, XLVIII; Petrie, 1933b: pl. LVII; Petrie, 1934a, pl. LX–LXIII; Petrie *et al.*, 1952: pls XXXII–XXXV for the stratigraphic relationship between many of these tombs and their surrounding architecture, see Kempinski, 1983: 135–136). The Swedish-Palestinian excavations have added a further five intramural tombs to this list ( Fischer, 2021: 156–157).

There were some unfortunate administrative errors in tomb numbering at ʿAjjul over the years. Firstly, the 600-series tombs were renumbered ‘Tombs 1–40’ for publication. ^3^ Secondly, numbers 200, 2000, and 2100 were assigned to actual tombs, rather than being reserved for recording surface finds from that ‘cemetery’ area (as was usual at both Fara and ʿAjjul). Finally, tomb numbers in the 400 series (dug in the first field season), and 1500 series (dug in the second field season), were accidentally reassigned to completely new tombs in the third and fourth field seasons, leading to a duplication of numbers used. Researchers need to be aware of these issues; fortunately, the duplicate sets were dug in different years and different parts of the site, so can be differentiated from each other through reference to the site publications.

## 4. Field recovery and recording methods

By the time he came to work in Palestine, Petrie was taking a less active role in day-to-day field supervision, relying heavily on the expertise of key staff members such as Leslie Starkey, who served as acting director of the dig whenever Petrie was absent. This included the entire first and third field seasons at Tell Fara, when Petrie spent the winter in Italy on the advice of his doctor ( Drower, 1995: 370, 375). Their workforce was primarily drawn from Bedouin families in the Wadi Ghazzeh region, with a handful of experienced excavators brought in from Egypt ( Drower, 1995: 365–366; Sparks, 2013b: 146–147). Unlike other foreign projects at the time, these Egyptian staff were not used as *reises* to oversee fieldwork ( Badè, 1934: 20, 22; Cline, 2022: 21); that role was reserved for the foreign members of the team. Rather, they were used to train the local workers, and brought in to excavate deposits where a more experienced hand was needed ( Petrie, 1927a: 5). Both Palestinians and Egyptians were deployed to clear out the contents of tombs, usually working in pairs where space permitted, with children given the job of sifting the spoil they produced for stray finds. A member of the English-speaking staff would be on hand to record the burials and tomb contents. In addition to documenting whatever was found, they were responsible for overseeing all the workers assigned to the cemetery area — sometimes upwards of 50 people ( Green & Henry, 2021: 67). The site surveyor — another westerner — might also be drafted in to make more detailed plans of the remains, and it is possible to identify individual work of this kind, thanks to the draughtsman’s initials appearing on the published plates (e.g.: G.F. Royds, published in Petrie, 1932a, pls LIII–LIV; Carl Pape, published in Petrie, 1933b: pl. XLIX). These also show that Petrie sometimes planned skeletal human remains himself (e.g., Petrie 1933b, pl. XLIX).

### 4.1 Burial conditions and the degree of in situ survival

The condition of the burials varied, depending on factors such as the nature of the surrounding soil and the degree of disturbance. Disturbances were recorded for at least 20% of the tombs discovered at ʿAjjul and Fara, and the actual figure may have been higher than this. As Price-Williams has noted, it isn’t usually recorded what had caused each disturbance ( 1977: 7); possibilities include tomb robbery, roof collapse, and interference from ancient building or additional grave cuttings. Although not all skeletal human remains were sufficiently well preserved for collection, such bones could sometimes be measured *in situ* ( Petrie, 1924: 2; Petrie, 1931b: 39). Where no bones were evident at all, this was usually noted.

### 4.2 Recording the dead

**Figure 2.**
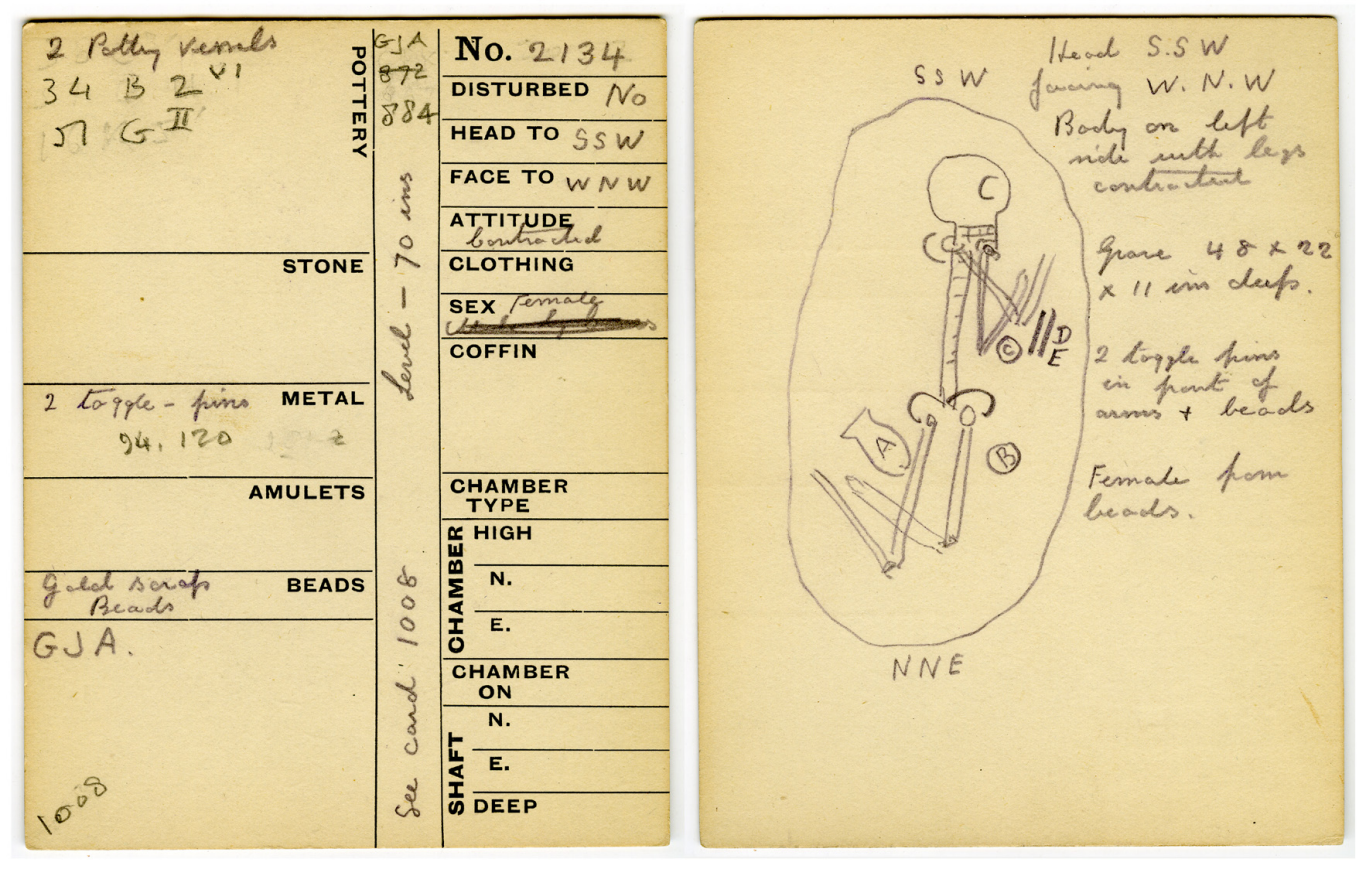
Tell el-ʿAjjul Tomb Card 2134. The front of the card summarises associated vessels and objects, locates the tomb, and describes the position of the deceased. The back provides a sketch and further description. The handwriting seems to be that of 1938 co-director Ernst Mackay. This image has been reproduced with permission from the UCL Institute of Archaeology Collections (Petrie Palestine Archive).

Tombs were formally recorded on special pre-printed cards ( Drower, 1995: 389; Sparks, 2013b: 156, Figure 9; see Figure 2). A selection of tombs were then chosen for publication, with the data summarised in tabular form ( Macdonald *et al.* 1932, pls XC–XCIII; Petrie, 1930a: pls XV, LXVIII–LXXI; Petrie, 1931a: pls LIX–LXI; Petrie, 1932a, pls LVI–LIX; Petrie, 1933b, pl. L; Petrie, 1934a, pls LXVI–LXVIII; Petrie *et al.*, 1952: pls XL–XLI). This process sometimes led to errors; as noted by Price-Williams ( 1977: 4) the tomb cards are usually the most reliable source of information.

Petrie described these cards as follows:

For tomb registers it is best to have cards, with printed title for every observation that should be made; usually sketch plans are put on the back of the card. Such cards are the size of a usual envelope, and stiff to keep flat in the pocket; two or more can be used for complex cases ( Petrie, 1934b: 120).

This was a system he had been using for many years, building on early types of tomb recording proformas used on Egypt Exploration Fund projects from Naville’s work at Abydos in 1908/9 onwards ( Quirke, 2010: 170, 186; Serpico, 2008: 5). Looking at material in the Petrie Museum of Egyptology archives, Petrie appears to have first introduced his version of the tomb card in his work at Tarkhan/Kfar Ammar in 1912. What Petrie added to this system was greater emphasis on recording and categorising the features of a burial and its associated finds. This was probably intended to assist his subsequent publication of the material — many of Petrie’s working practices seem to be designed with this in mind.

The idea was that this card would act as a prompt to the excavator, ensuring all essential information would be recorded. Petrie’s views on what was essential are outlined in his 1904 book, *Methods and Aims in Archaeology,* a field manual that offered advice on all aspects of running an excavation. He recommended recording the following:

Position relative to other tombs. Size of pit, direction, depth. Position of chamber. Filling intact, or estimate of time that it has stood open anciently by the weathering of the sides. Objects found loose in filling. Chamber plan. Primary or secondary burial. Position of body, head direction, face direction, attitude of body and limbs. Position of beads and small objects on body. Note if beads follow any pattern or order; record order of as long groups of beads as possible for rethreading; wrappings, amount and nature. Coffin or cartonnage; inscription and figures, if any ... Skull and jaw to be removed for measurement ... Position and nature of all offerings and objects placed in the tomb ( Petrie, 1904: 52–53).

In reality, the tomb cards used in Palestine were designed to record only some of this information, and while their format changed slightly over time, they were not modified specifically for use with Palestinian, rather than Egyptian burials. As a result, the cards retained somewhat inappropriate headings for recording clothing, which was never preserved, and coffins, which were extremely rare. It is also interesting that, in 1904, Petrie was not yet advocating recording the sex of the individual on these cards, although the cards later came to include a separate section for this information. Despite this, it is clear that these cards served as a record of primarily *archaeological* information. Tomb card design did not allow for detailed measurement of the individual, or records of dentition or pathology — probably reflecting Petrie’s expectations as to who would be filling these cards out.

While Petrie’s tomb cards did encourage a certain amount of consistency, allowing for cross-context comparisons, different field recorders developed their own styles of completing the cards, with varying degrees of detail provided, and some types of information omitted altogether. The lack of pre-printed heading prompts on the back of the cards in particular led to a degree of inconsistency in the way this section was completed; on some tomb cards there is useful logistical information being provided on things such as excavation date, workmen’s names, the amount of *bakshish* (bonuses) paid, and sketches of tomb plans, sections, or location details. Other excavators simply left the back of the card blank. And while information concerning tomb disturbance, attitude and orientation of the body, burial cut/chamber dimensions, and associated finds was usually recorded, where available, the evidence was not always there to be noted. Consequently, any sort of statistical analysis of trends across cemeteries or graves of a particular period has been somewhat restricted by gaps in the data (as noted by Braunstein, 1998: 9).

**Figure 3.**
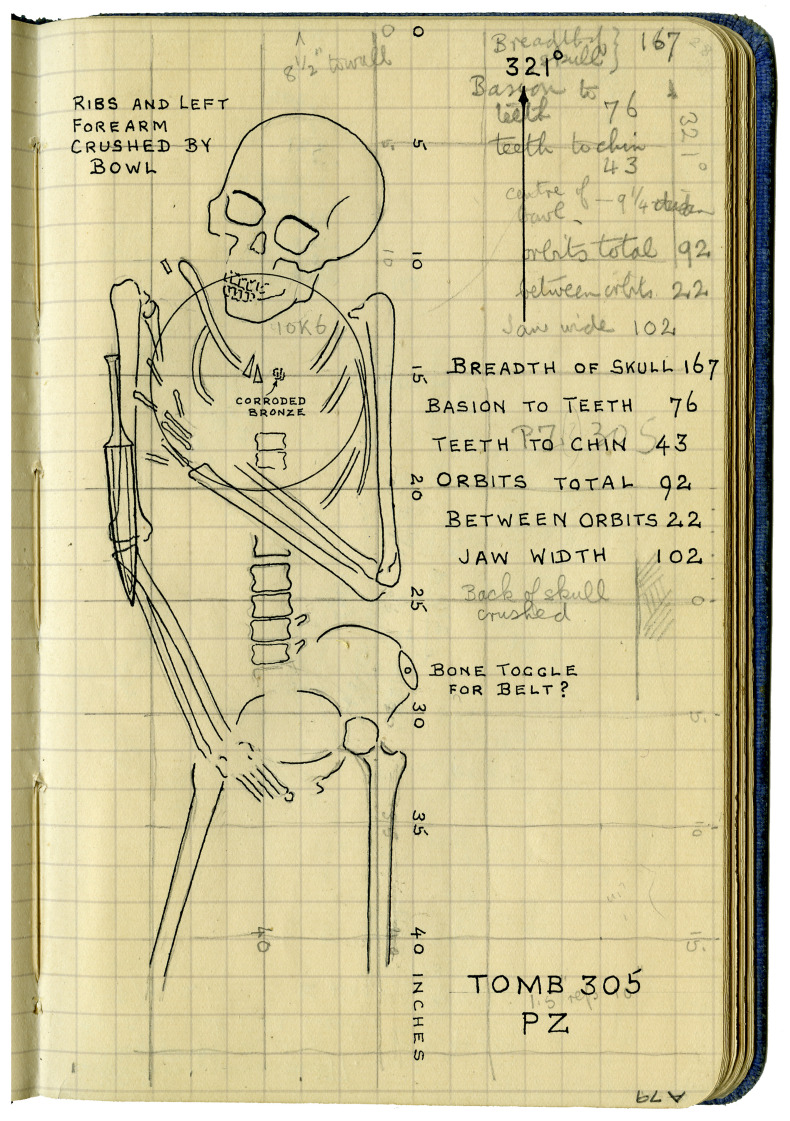
Carl Pape’s plan and measurements of the skeleton from ʿAjjul Tomb 305 ( Pape, 1933: A79). None of this information appeared on the field card for this tomb, but the plan, bearing Pape’s initials, was published in *Ancient Gaza III* ( Petrie, 1933b: pl. XLIX). This image has been reproduced with permission from the UCL Institute of Archaeology Collections (Petrie Palestine Archive).

Pre-printed tomb cards were sometimes not available, and so substitutes were created using notepaper or blank postcards. Supervisors would also occasionally record tomb information in their field notebooks. For example, in the fourth field season at ʿAjjul, six loose pages from ‘Mr Peckham’s notebook’ are inserted alongside the card for Tomb 1502 (published Petrie, 1934a, pl. LIX), Anne Fuller makes reference to ‘my notebook’ on the card for Tomb 1816, and similarly, Wu Gin Din refers to five pages with more details of Tomb 1928 in 'my notebook.’ Unfortunately, most of these field notebooks have since been lost or discarded. These lost notebooks are presumably an additional source of information that is unattested on the tomb cards themselves but which appears in the published record. This is borne out by the few notebooks that have survived: those used by Petrie throughout his time in Palestine, and those of his field surveyor in the later seasons at ʿAjjul, Carl Pape. These are now held in the Institute of Archaeology Petrie Palestine Archive. They were clearly not intended as a permanent record, but take the form of informal working notes used to calculate levels and triangulations, record lists of workmen or finds, sketch plans, and on occasion, document tomb contents or skeletal measurements ( Petrie, 1924: 2; see Figure 3).

Petrie had exhibited an interest in biometrics from early on in his career (e.g., Petrie, 1894: 3), and he frequently wrote about the importance of measuring skeletal human remains, including skulls (e.g., Petrie, 1897: 25–30; Petrie, 1904: 53). It is clear that measurements were collected for some of his Palestinian remains ( Petrie, 1932a: 5; Petrie, 1931c). Yet the tomb cards make no provision for recording this information, and actual measurements appear on the cards only rarely (2.3% of ʿAjjul tomb cards, and 0.2% at Fara; see Table 3). This data was never systematically published, although a summary table outlining various skull measurements appeared in the final ʿAjjul report ( Petrie *et al.*, 1952: pl. XXXI). This was drawn up by Petrie, whose initials appear at the bottom of the published plate, and published posthumously, which may explain why it is presented without any accompanying discussion.

**Table 3.** Tombs recording osteological data. Counts are based on extant tomb cards only, and exclude missing or cancelled cards.

	Sex data	Age data	Anthropometric data	Use of anatomical terminology	Total tombs excavated
Tell el-ʿAjjul	130	29	30	34	1300
Tell Fara	35	0	1	1	488
Total	165	29	31	35	1788
% of total tombs dug	9.2%	1.6%	1.7%	2%	

The tomb cards did make provision for recording biological sex, but this information was suggested for only 7.1% of the burials dug at Tell Fara, and 10% of those from ʿAjjul. Age was recorded less frequently: for none of the Fara burials, and only 2.2% of those from ʿAjjul ( Table 3). Overall, one gets the impression that there was either less interest in recording this type of information at the point of excavation at Fara than ʿAjjul, or a lack of available expertise. It would also seem that the tomb cards were *not* intended to be the primary means of recording osteological information, and such details as appeared were added in an opportunistic manner. This is borne out by a season-by-season analysis of the data, which shows that for most seasons, sex, age, anatomical terminology and anthropometric data are exceedingly rare. The exceptions are the fourth and fifth seasons at ʿAjjul, which suggest someone with this knowledge being present as burials were being dug and recorded.

### 4.3 Who recorded the dead?

The question of who captured skeletal information in the field is key to determining the accuracy of the information recorded, which will be discussed further below. Unfortunately, the tomb cards almost never include the name of the person recording them, and while one can do a certain amount of detective work studying the handwriting involved, it has not yet been possible to definitively identify all those responsible for filling out specific records. The most one can do is identify who may have worked in the cemeteries in particular season, and consider what is known about their levels of anatomical training or expertise. A summary of this information is presented in Table 4.

**Table 4.** Season-by-season list of excavated tombs. A list of field staff likely to have been involved in either planning tombs, or supervising and recording cemetery work. *Confirmed cemetery supervisors; +Confirmed tomb planners.

Field season	Tombs excavated	Field staff likely to be involved in tomb recording	Medical/anatomically-trained staff	Notes
Jemmeh	Bronze Age, Roman and ‘Medieval’ tombs	W.M.F. Petrie (director) G.L. Harding D.L. Risdon J.L. Starkey	Dr G. Parker	No surviving field records; no skeletal information published.
Fara 1	T. 101–132 T. 201–272 T. 501–533 T. 601–654 T. 701–704	J.L. Starkey (director) G.L. Harding * D.L. Risdon O. Tufnell *		Tufnell is known to have worked in the 100 and 200 cemeteries.
Fara 2	T. 133–138 T. 534–596 T. 655–661 T. 705–763 T. 801–865 T. 901–904	W.M.F. Petrie + (director) H.D. Colt G.L. Harding * O. Myers J.L. Starkey * O. Tufnell *	Dr G. Parker	
Fara 3	T. 139 T. 662–663 T. 905–978 T. 1001–1027	J.L. Starkey (director) H.D. Colt G.L. Harding + O. Myers		Harding plans tombs in 1000 Cemetery.
Fara 4	T. 979–985	W.M.F. Petrie (director) G.L. Harding N. Scott O. Tufnell		Overlapped with Ajjul season 1.
ʿAjjul 1	T. 101–199 T. 200–298 T. 401–445 T. 601–640 T. 801–812	W.M.F. Petrie (director) H.D. Colt G.L. Harding J.L. Starkey O. Tufnell * J.G. Vernon G.F. Royds (surveyor) *	Dr G. Parker	Petrie commented that a large mass of bones near the tunnel entrance found this season had been ‘left for an anatomist to separate’ (Petrie 1931: 4), implying there was no one present on the dig with the skills to do this.
ʿAjjul 2	T. 1001–1099 T. 1100–1170 T. 1401–1451 T. 1501–1575	W.M.F. Petrie + (director) N.P. Clarke G.L. Harding * W. Hastings T.P. O’Brien + J.L. Starkey O. Tufnell * G.F. Royds + (surveyor)	Dr Sperrin Johnson +	
ʿAjjul 3	T. 301–399 T. 401–420	W.M.F. Petrie + (director) H. Falconer G. Maconachie N. Wheeler G.F. Royds (surveyor) C. Pape (architect) +		400 tomb numbers accidentally reused for a new set of graves in the Lower Cemetery.
ʿAjjul 4	T. 420A–499 T. 501–508 T. 1203–1299 T. 1301–1354 T. 1452–1499 T. 1501–1559 T. 1601–1699 T. 1701–1782 T. 1800–1863 T. 1901–1971	W.M.F. Petrie (director) H.E. Bird * Wu Gin Ding * A. Fuller * C. Peckham * J.R. Stewart * N. Wheeler * + Carl Pape (surveyor) +		1500 tomb numbers accidentally reused for a new set of intramural burials; Tombs 421–432 accidentally reused for intramural burials in area J and elsewhere. Bird excavated the 400 and possibly the 1700–series tombs. Ding dug the 1200 and 1900-series tombs. Fuller dug the 1600 and 1800-series tombs. Peckham worked on the 1500-series tombs Wheeler excavated the 400 and 1500-series tombs.
Ajjul 5	T. 1972–1999 T. 2000–2099 T. 2100–2151	E.J.H. Mackay (director) M.A. Murray (director) L. Kiralfy W.M.F. Petrie C. Pape (surveyor)		All tombs were intramural, located in Area G.

Anatomical training, including techniques for measuring skeletal human remains, was considered to be part of the essential training of a ‘Petrie’ archaeologist, and so was taught as part of the curriculum for Margaret Murray’s Certificate in Egyptology at University College ( Murray, 1961: 12). Anatomy lectures were given by Douglas Derry (Professor of Anatomy), Geoffrey Morant, and later on, by Elliott Smith, Egyptology graduate and medical doctor Edith Guest, and then Neurology lecturer Una Fielding ( Janssen, 1992: 12, 24; University of London, 1919; University of London, 1920; University of London, 1925; University of London, 1936). Any students who had been through this process should have possessed the necessary skills to accurately record the human remains at these sites. Murray would also loan students bones to study from ‘George,’ a human skeleton she had purchased as a teaching aide ( Janssen, 1992: 25). While some of Petrie’s field staff in Palestine had learned hieroglyphs under Murray, including Starkey (John Starkey pers. comm. 27/10/2006), Harding ( Tufnell, 1980b: iii) and Risdon ( Petrie, 1927b), they do not appear to have taken the full Egyptology training course, so we don’t know how much opportunity they had to pick up skills in measuring or identifying skeletal remains. They may, however, have acquired experience in this elsewhere.

**Figure 4.**
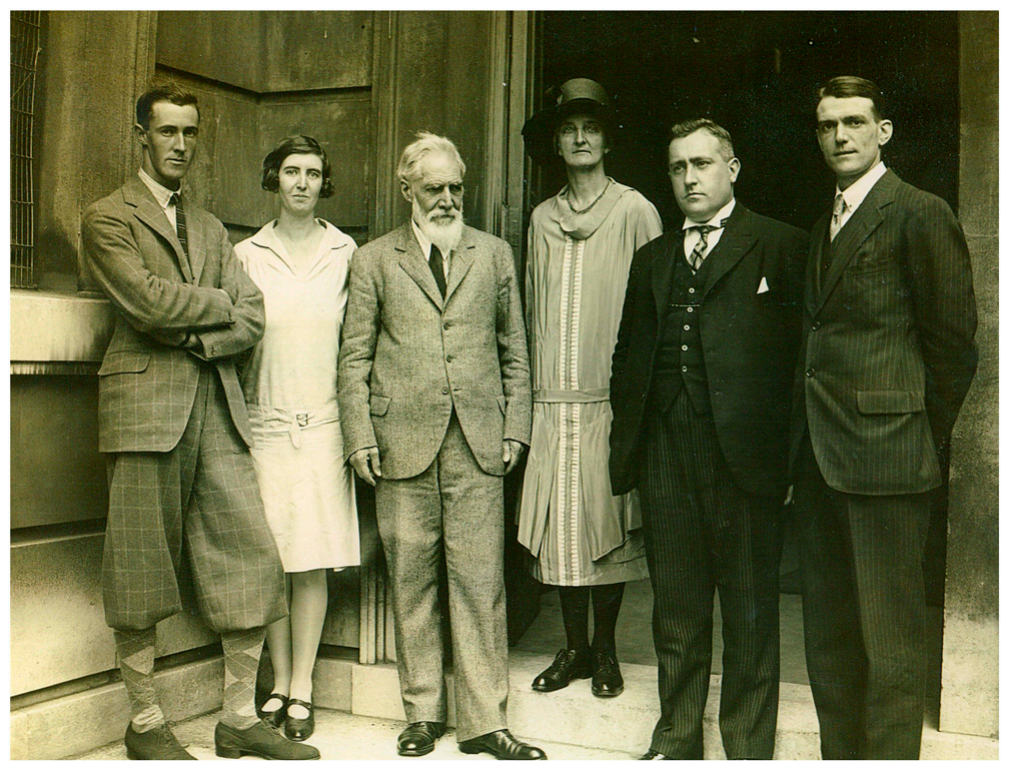
Petrie’s excavation team at University College in the late 1920s. Left to right: Gerald Harding, Olga Tufnell, Flinders Petrie, Hilda Petrie, Leslie Starkey and Denziloe Risdon. Photograph from the Starkey family archive, reproduced with permission from Wendy Slaninka.

This would appear to have been the case with Leslie Starkey who was Petrie’s right-hand man for a number of years, and directly responsible for supervising the field staff ( Figure 4). Prior to coming to Palestine, he had been involved in recording tombs at Qau in Egypt in 1923 and 1924, after first receiving guidance on sexing skeletal human remains from Professor Derry ( Brunton, 1927: 1, 5). Dig director Brunton had himself been trained in anatomy by Derry back at University College, and so would also have been on hand to advise ( Sheppard, 2010: 178), although he appears to have let his field staff do much of the sexing of the Qau burials ( Brunton, 1927: 5). In theory, Starkey would have been able to provide identifications himself, as well as passing on his knowledge to less experienced colleagues — although it should be pointed out that a few pointers from an 'expert' who was only at the site for four days would not in itself be sufficient to make Starkey an expert himself.

**Figure 5.**
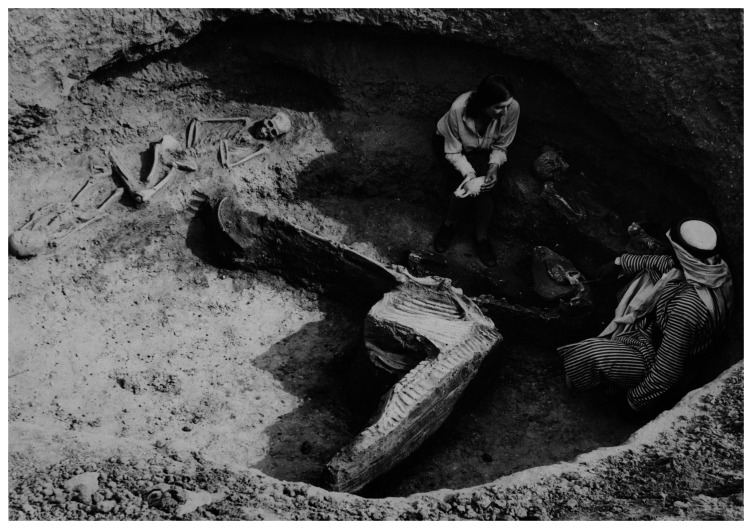
Olga Tufnell and an unidentified workman excavating Tell el-ʿAjjul Eastern Cemetery Tomb 411. This tomb contained three human burials and an equid skeleton. This image has been reproduced with permission from the UCL Institute of Archaeology Collections (Petrie Palestine Archive).

Starkey’s role on the dig was one of oversight and management, but he was sometimes also involved in cemetery supervision ( Petrie, 1928b: 8). Generally, however, this work was assigned to other members of the field team. One such was Olga Tufnell, who was given the job of supervising excavations in the 100 and 200 cemeteries at Tell Fara ( Figure 4– Figure 5). Petrie was so impressed with her work, he allowed her to write this material up in *Beth Pelet I* ( Tufnell, 1930). This was the first time she had excavated in the field; previous experience had consisted of working as Assistant Secretary to the BSAE in London, mending pottery, and assisting with the annual exhibition there, followed by a short season recording tomb reliefs at Qau ( Green & Henry, 2021: 7, 31; Henry, 1985: 2). None of this would have prepared her for her new role at Tell Fara, and it seems there was little training on offer either. ‘We were all in at the deep end; we had no experience of how you recorded a tomb. He [Starkey] had his cards made out and we filled them in as best we could. And that was that’ ( Tufnell, 1985: 6; see also Green & Henry, 2021: 8). She was joined in the cemeteries that season by Gerald Harding, who was similarly inexperienced ( Figure 4). Both continued to play a key role in excavating burials in future Fara field seasons. This lack of specialist training would seem to be borne out by the Fara tomb cards, which do not show evidence for the application of anatomical expertise.

Another important staff member in the early years was Denziloe Risdon ( Figure 4), a retired naval commander who joined them at Tell Jemmeh and for the first Fara season. He acted as a field supervisor and sometime surveyor ( Green & Henry, 2021: 70, 85); it is not known if he dug or planned any tombs at either site. Petrie mentions that Risdon had studied hieroglyphs under Margaret Murray ( Petrie, 1927b); however he is not listed as a day or evening student in the university calendar, so may have been taught privately by her. Risdon did subsequently enrol in an archaeology degree at University College ( University of London, 1929: 477) and went on to research and publish a large group of skeletal human remains from the Palestinian site of Lachish, financed by the Crewdson Benington studentship at UCL’s Biometric Laboratory ( Risdon, 1937; Risdon, 1939). This work was done under Morant’s supervision at this laboratory over a period of three years ( Risdon, 1939: 100). This later interest means that Risdon could potentially have been a source of specialist knowledge for human remains while at Fara, and yet there are no signs of particular osteological expertise evident for the tomb cards for the single season he was there. Thus, while Risdon went on to gain significant specialist knowledge of osteology, he may have gained this only after he finished working for Petrie.

Various other staff members came and went on the excavations; of these, Noel Wheeler, Anne Fuller, Wu Gin Ding, James Stewart and Cyril Peckham are all known to have supervised fieldwork in the cemetery. Wheeler probably had the greatest level of experience with this type of work, having previously excavated numerous burials at Qau and Gizeh in Egypt ( Brunton, 1927: 1; Crowfoot, 1943: 126). In the final field season at ʿAjjul, it is suspected the two official field directors, Margaret Murray and Ernst Mackay, would also have been able to bring some expertise to bear on recording osteological data — and the latter may be behind the atypically high rate of age-data provided on the tomb cards from this season. Carl Pape, who was site architect and surveyor in the final three seasons at ʿAjjul, also made detailed plans of many of the burials — and sometimes annotates these with technical terminology that suggests a certain degree of familiarity with skeletal anatomy (e.g.: Pape, 1933: A63, A79, A87–88).

Finally, of course, there was Petrie himself, who often recorded anthropometric data in his own notebooks and planned some of the tombs himself, as noted above. This was a skill he seems to have acquired early in his career; in a letter to Karl Pearson back in 1894 Petrie requests a ‘published diagram of the exact positions of standard measurements’ so he can measure skulls *in situ,* when they proved unsuitable for lifting ( Petrie, 1894: 3). He also tells us that he collected the anthropometric data from his Deshasheh skeletal human remains himself ( Petrie, 1897: 27).

Occasionally, more expert knowledge appears to have been available. We know, for example, that Petrie’s staff at Tell Jemmeh, Fara season 2 and ʿAjjul season 1 included a medical doctor, George Parker, and although we are not told that he did any particular work on the human remains, it is possible that he contributed some advice or identifications ( Parker, 1927). Then in the second field season at ʿAjjul Petrie was joined by Dr Sperrin-Johnson, a biology Professor with training in anatomy who had just come from a post at University College Auckland, and who was given the job of measuring skulls ( Drower, 1995: 387; University College Cork, 2020). As his presence did not correspond to an increase in the amount of osteological biometrics recorded on the tomb cards for that season, Sperrin-Johnson presumably kept his own records of this work. His initials appear alongside those of the surveyor G. Royds on one published plan of a mixed group of human and animal bone, where individual elements have been identified ( Petrie, 1932a: pl. L). We can note here that, even when experts were available, Petrie never seems to have adopted a formal card system for recording specialised anatomical or craniometric data, even though such systems had been previously adopted by some of his contemporaries — notably on the Archaeological Survey of Nubia in 1908 ( Cockitt, 2014: 15–16, Figure 1).

In subsequent seasons, there are a few occasions where specialised terminology was employed to record some of the burials. For example, the back of the card for Tomb 313, excavated in season 3 at ʿAjjul, reads:

Room in Town P [Tomb] 313. [...] Head lying at East. Orientation N 130 E. Anthropological meas. (‘Petrie’) & Notes. Cranial bone broken. Dolichocephalic — narrow frontal region considerable widening of parietal region. Teeth well preserved; second molar only slightly worn. Age not more than 40 years. Strongly developed superciliary protuberance and air-sinuses. Measurements: Humerus right 12.25, left —, Radius right 10.45, left —, femur right 16.20, left 16.20. Stature by form[ula] 158.66 cms. 62.5 in[che]s.

This indicates that there was someone present at the site that season with a good level of anatomical knowledge; their handwriting has, sadly, not yet been identified. It is however noteworthy that the skeletal human remains in this particular series of burials at ʿAjjul were generally better recorded, with several sketches of them by Pape and Petrie appearing in the third season report ( Petrie, 1933a: B11–12, B15; Pape, 1933: A59, A67, A79, A83–4, A87–8; Petrie, 1933b, pl. XLIX; see Figure 3).

### 4.4 Field recovery, preservative treatments and marking

Petrie never states what his retention policy was for the human remains he excavated in Palestine. Earlier in his career, however, he had suggested that complete skeletal remains should only be preserved if they were from 'rarer periods' ( Petrie, 1904: 53), suggesting their importance was in direct proportion to his interest in the material. He also recommended that the skull and jaw should be taken 'for measurement' ( Petrie, 1904: 53; see also Petrie, 1914: 2) — a reminder of Petrie’s underlying interest in craniometric studies. These allude to some selectivity in what was removed from the field, reflecting also the type of material that has survived from his Palestinian excavations; predominantly skulls. The available assemblage may also have been subject to an additional bias as the majority of skeletal human remains exported to the United Kingdom were included in Karl Pearson’s Eugenics Department’s collection of reference skulls at University College in London (see Section 6.2 below).

This is further supported by Petrie’s treatment of the skulls, which were block-lifted and treated in the field to facilitate transportation without further cleaning.

In the store room they [the skulls] were immersed in melted wax for a time, to soak in and expel the air. When cold they were completely swathed in a tight strip of muslin, and finally waxed to secure this binding. Nothing short of this will preserve, in transport, skulls full of earth such as these ( Petrie, 1932a: 5).

**Figure 6.**
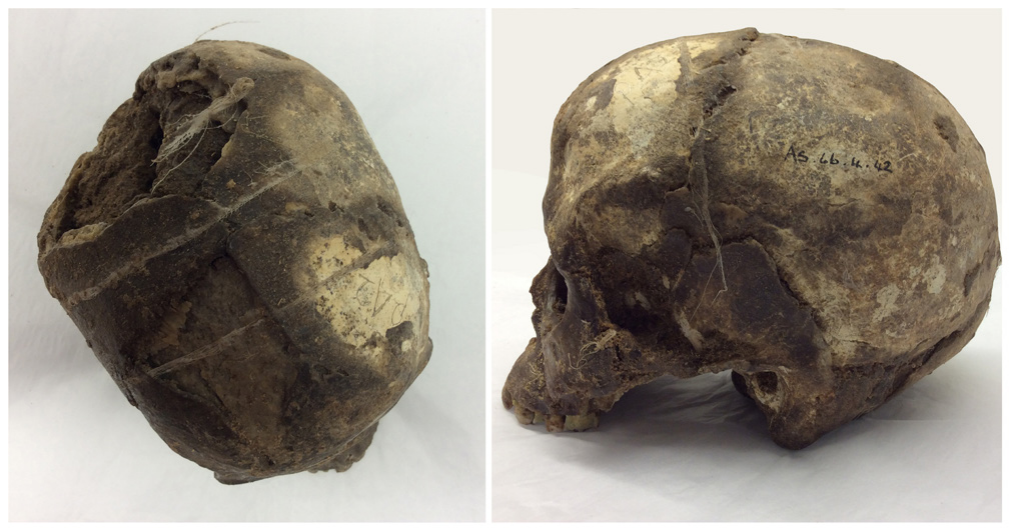
Top view (left) and side profile (right) of skull As.66.4.42 from Tell el-ʿAjjul area MU level 1090.’ This shows Pearson’s catalogue number marking (P42), wax coating, and traces of muslin binding. The interior is still filled with excavation soil. Photographs by Rachael Sparks, reproduced with permission from the Duckworth Laboratory.

Several skulls and long bones from all three of his Palestinian sites, now in the Duckworth Collection, show traces of these paraffin wax and muslin bindings ( Figure 6). Many are still full of soil from their original contexts. Contemporary film footage from Lachish, another Palestinian site where Starkey began his own excavations in 1932, shows wax was also applied to skeletal human remains *in situ* ( O’Grady, 2017; O’Grady, 2019: 63–64, Figure 2). A similar method was being used to preserve the skeletal remains uncovered in 1920s excavations at Kish in Iraq and elsewhere ( Buxton & Rice, 1931: 58; Gedye, 1947: 10; O’Grady, 2019: 62–63).

Petrie adopted waxing as a selective treatment for skulls considered to be in ‘passable’ condition ( Petrie, 1931b: 39); the ʿAjjul and Fara tomb cards mention some 43 skulls being given this treatment, although the actual figure is likely to have been much higher. Waxing skulls in their down-time was one of the tasks allocated to staff members like Olga Tufnell and Oliver Myers ( Green & Henry, 2021: 72, 85, 178). Petrie had used similar techniques in his earlier digs in Egypt ( Drower, 1995: 324; Petrie *et al.*, 1925: 9; Petrie, 1930b: 226) and many of his contemporaries did the same ( O’Grady, 2017; O’Grady, 2019: 62–64). Later, at Lachish cellulose and shellac were also employed as preservative treatments ( Inge, 1938), the latter recommended for being easier to remove than wax ( Gedye, 1947: 10). Occasionally other bones were also waxed (e.g.: Fara Tomb 133, ʿAjjul Tombs 407 and 2009), or desalinated ( Petrie, 1930b: 128). It would appear that measurements were often taken before ‘paraffining’ took place ( Petrie, 1924: 2), but where material was sent elsewhere for later study, these coatings had to be removed before the material could be studied ( Risdon, 1937). Removing this wax sometimes proved problematic, and its negative impact on future use has been noted ( Buxton & Rice, 1931: 58; O’Grady, 2019: 61, 66, 70).

**Figure 7.**
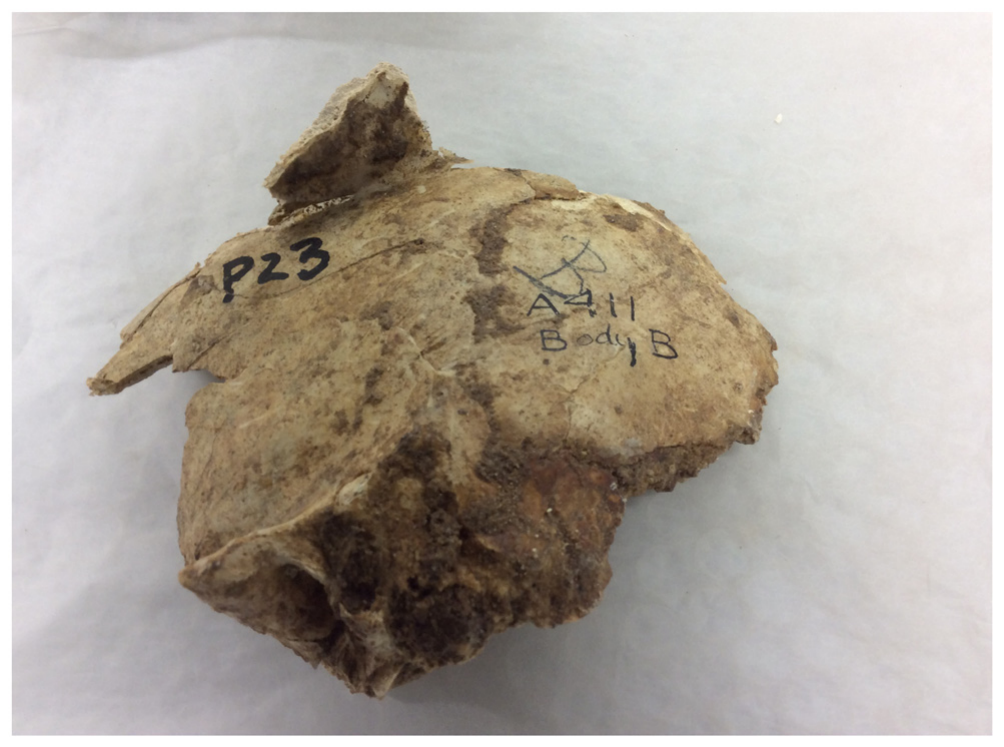
Skull As.66.4.23 from Tell el-ʿAjjul Tomb 411, excavated in the first field season. This shows Petrie’s original field markings in pencil (‘B’) and ink (‘A411 Body B’) and Pearson’s laboratory number (‘P23’). Photograph by Rachael Sparks, reproduced with permission from the Duckworth Laboratory.

Earlier in his career, Petrie had advocated marking every human bone in 'China ink’ and in a consistent place ( Petrie, 1895; Petrie, 1904: 51–52), which in itself implies that he saw a value in retaining some of this type of material for future study. While some of the Petrie Palestinian bones and skulls in the Duckworth Laboratory bear ink or pencil field markings (e.g.: Figure 7), often underneath their bindings or wax treatments, many do not. This lack of markings will be addressed in Section 7.2.

## 5. Disposition of the human remains

Petrie excavated over 2000 ancient tombs, several of which contained multiple interments, and an unknown number of later ones. And yet, as will be seen, the whereabouts of most of the human skeletal remains recovered from these cemeteries are currently unknown. While the Tomb Cards suggest that some bodies were too poorly preserved to record, and so were probably discarded in the field, this still leaves many individuals unaccounted for.

One possibility is that the human remains were reburied after excavation. Petrie does mention reburying skeletons at an earlier dig at Abydos, once he had collected the necessary anthropometric measurements, although he retained the associated skulls ( Petrie *et al.*, 1925: 9). However, this did not seem to be his normal practice, and he did not record reburying any of the skeletal human remains from Palestine.

We do know that some osteological material was sent to England for further study. This included cremation remains from Fara Cemetery 200, which he sent to the eugenicist and statistician Karl Pearson. Pearson’s conclusions were presented in the first Fara publication. Pearson’s report is extremely short — some 209 words — consisting of brief introduction, followed by identifications of the age and sex of the individuals, with a few additional comments, such as ‘Sutures closed internally. Female? circa 40,’ or ‘Adult, probably male. Two teeth very much worn down’ ( Petrie, 1930a: 13). Despite its brevity, he still found room to comment on the supposed race of the group, who were characterised as ‘small’ (ibid). The current whereabouts of this cremation material is unknown.

Petrie also donated numerous skulls to Pearson’s reference collection in the Eugenics Department at University College in London ( Petrie, 1932a: 5). While Pearson or others might have reported back to Petrie with identifications or comments on this material, they are not credited with doing so in any of the subsequent excavation reports. This is the material that was subsequently transferred to the Duckworth Laboratory, which represents around 127 individuals; it will be discussed in more detail below.

A few small fragments of human bone, including phalanges, were also sent to London by accident, accompanying finger rings and other small finds from the Tell Fara and Tell el-‘Ajjul tombs. Some of these are now in the UCL Institute of Archaeology Collections, and its possible that others exist elsewhere. These survived incidentally, rather than through any deliberate process of curation, and have never been formally studied.

The remainder — and majority — of the human skeletal remains from these excavations must therefore have remained in Palestine. It is known that many skulls were still being stored at the ʿAjjul dighouse as late as 1935, in a room known to the dig team as the ‘skullery’ ( Green & Henry, 2021: 72). This was sometimes used as an impromptu bedroom for dighouse visitors ( Green & Henry, 2021: 93), and Veronica Seton-Williams reported hearing lizards knocking skulls off its shelves during her short stay here en-route down to excavations in Sinai ( Drower, 1995: 410). The dighouse was not in use at the time. The last ʿAjjul field season took place from February to April 1938 ( Drower, 1995: 415; Hamilton, 1938). Then in September of that year, the ‘Ajjul dighouse was looted and burned down, destroying its contents ( Drower, 1995: 416). If there were any skulls still stored there at this time, they were presumably destroyed as well.

One final possibility is that some of the human remains from these excavations were transferred to the Department of Antiquities in Jerusalem at the end of each field season. However, no documentary proof of a transfer has been located. Osteological remains do not feature in the official end of season documentation, which only includes information about the antiquities discovered and their field contexts. It would also appear that this material has never been stored in the anthropological department of its successor, the Israel Department of Antiquities and Museums (now the Israel Antiquities Authority). While some human remains were reburied in the 1990s at the request of the religious parties, they did not include any from Petrie’s excavations (Y. Nagar pers. comm. 2023).

## 6. The intellectual rationale behind collecting skulls and anthropometric data

While Petrie understood the value of aspects such as sex and age-at-death for interpreting his burial evidence, he was also looking to use human remains as a way of identifying populations through the collection of anthropometric data ( Challis, 2013: 178, 195; Petrie & Quibell, 1896: 51–54, 59–64; Petrie, 1906; Ramsey, 2004: 16). This focus makes sense when one considers Petrie’s lifetime interest in mathematics and measurement — reflected in his pyramid-measuring activities at the start of his Egyptological career ( Dash, 2017; Drower, 1995: 34–63), his development of the technique of seriation ( Challis, 2013: 170; Gertzen & Grötschel, 2012), and his long-standing interest in weights and measures ( Drower, 1995: 68, 128, 252; Petrie, 1926; Petrie, 1934c). But in his work with human skulls, Petrie aimed not just at quantification, but also to link physical characteristics to intelligence and place hierarchical values accordingly (e.g., Challis, 2016; Petrie, 1902; Petrie, 1907a:1–12).

At this point, it becomes essential to address a problem that underlies Petrie’s research: like many of his contemporaries, he operated under a colonial framework that coloured his perception of past people and cultures and influenced the way he used the data he collected. He aided in the collection of anthropometric data which was used to support eugenic research and a wider agenda of ‘racial improvement’ through his relationship with the polymath Sir Francis Galton and the statistician Professor Karl Pearson. This paper will now turn to exploring these relationships and demonstrate how Petrie was not only influenced by their work, but also contributed to it.

### 6.1. Flinders Petrie and Sir Francis Galton

The division of humans into groups based on physical attributes was of interest to many early anatomists and natural philosophers of the 18th and 19th centuries such as Linnaeus ( 1735; 1738), Blumenbach ( 1775; 1795), Camper (1791) and Verneau (1875). Blumenbach established the five human ‘varieties’ ( 1775), familiar to many even in today’s vernacular, which he later referred to as ‘races’ ( 1795), though it is important to highlight that he did not promote hierarchy within this system. His view was supported by Alexander von Humboldt ( 1858, translated by Elise C. Otte: 357):

While we [von Humboldt and Blumenbach] maintain the unity of the human species, we at the same time repel the depressing assumption of superior and inferior races of men.

At the time, ‘race’ could be used in a neutral tone, synonymous to a currently preferred term ‘population’. This was not, however, always the case. Unlike Blumenbach, Sir Francis Galton was a proponent of intellectual superiority of certain ‘races’ (e.g., Galton, 1892: v, x, 31). He was also a believer of hereditary intelligence and that the betterment of humanity could be achieved through selective breeding and statistics ( Galton, 1865a; Galton, 1865b; Galton, 1869; Galton, 1892: vii–xxvii, 31). Galton’s research included experiments with sweet peas that used computational methods; his ‘regression towards the mean’ and further work on mathematics have become essential to contemporary statistical research. His work eventually led him to coin the term ‘eugenics’ ( 1883: 17). For an analysis of contemporary 19th century reactions to his book *Hereditary Genius* ( 1869; 1892), see Gökyigit (1994).

Petrie appears to have been aware of Galton’s work from as early as 1878, when he wrote a letter to the editor of the journal *Nature,* responding to Galton’s paper on *Composite Portraits* ( Galton, 1878; Petrie, 1878). Petrie wrote to Galton again in 1880, several months before setting out for his first expedition to Egypt ( Drower, 1995: 68, 476–477). The aim of this expedition was to measure the Great Pyramid in order to prove the theories of Piazzi Smyth, a family friend ( Drower, 1995: 27–30), though his results ultimately had the opposite effect ( Challis, 2013: 79). On returning to England, Petrie discovered that the Royal Society had commissioned a similar survey, and decided to send them his work. Galton, at the time an active member of the Society, read his manuscript and was impressed enough to redirect the funding for their proposed survey to Petrie ( Challis, 2013: 79). This led to the publication of Petrie’s book, *The Pyramids and Temples of Giza,* in 1883 *,* and set Petrie and Galton up for a friendly working relationship that was to last for decades ( Challis, 2013: 81).

Like many of his contemporaries, Galton had begun measuring skulls to find correlations between shape and cognitive and personal qualities. Visual imagery was also being used as a way of exploring physiognomy, through photography of contemporary individuals ( Challis, 2013: 8–9, 75–6). In 1886, this was extended to include archaeological research, when Galton provided Petrie with the necessary funds to complete a commission for the British Association for the Advancement of Science (BAAS) to photograph profiles of people depicted in ancient Egyptian art ( Challis, 2013, 93; Drower, 1995: 106). The resulting report, *Racial Photographs from the Egyptian Pictures and Sculptures*, was submitted to the Association the following year ( Petrie, 1887), with copies being printed for the public on request ( Anon, 1888). This appears to have been Petrie’s first foray into data collection in support of eugenic research. 

From 1888, Petrie started to send skulls from his excavations to specialists for further anthropometric study, such as pathologist Rudolf Virchow ( Challis, 2013: 111), Alexander Macalister, Chair of Anatomy at the University of Cambridge ( Challis, 2013: 123), or Professor William Flower of the Natural History Museum ( Challis, 2013: 111). Later, he deposited skeletal remains with the American College at Asyut in Egypt ( Petrie, 1907b: 2), and in the Department of Ethnology at the British Museum ( Petrie *et al.*, 1910: 4, 16). And, as we’ve seen, he also appears to have engaged in the collection of anthropometric measurements in the field. For a summary of Petrie’s craniological research in Egypt, see Gaballah, el-Rakhawy and el-Eishi (1972).

### 6.2. Petrie, Karl Pearson and the Biometric Laboratory

**Figure 8.**
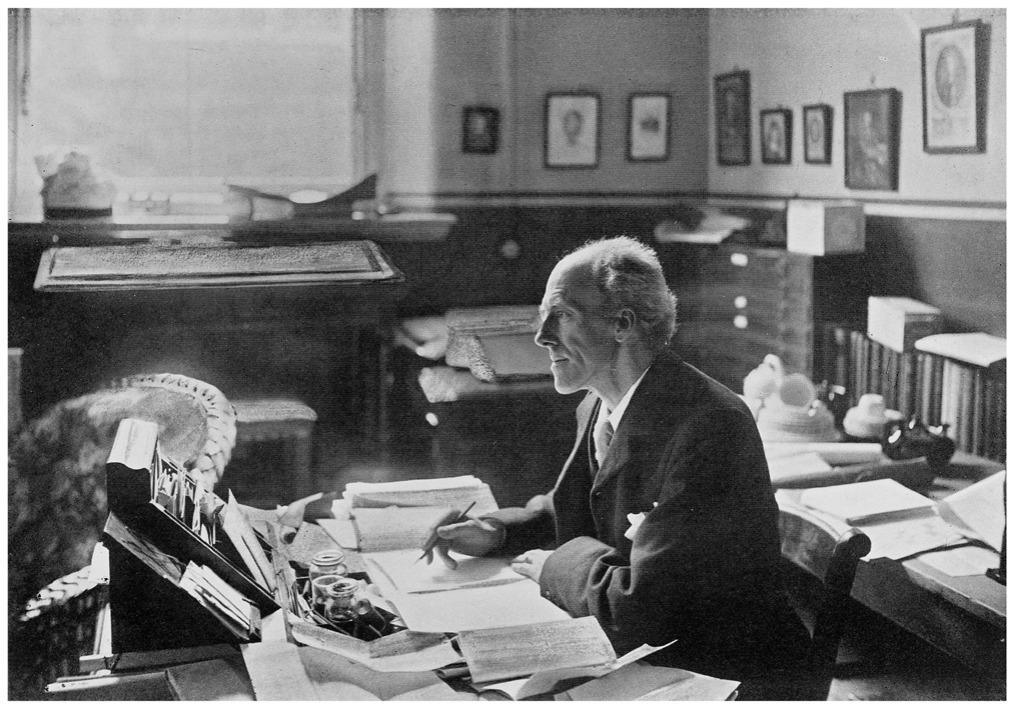
The biometrician Karl Pearson at his desk in 1910. Courtesy of the Wellcome Collection attribution 4.0 International (CC BY 4.0). Available at: < https://wellcomecollection.org/works/pexttqw4> [accessed 5 March 2022].

Another key figure connected to Petrie’s studies of skeletal human remains was the mathematician and statistician Karl Pearson (1857–1936; Figure 8). He became the Professor of Applied Mathematics and Mechanics at University College in 1884 and was appointed to a second professorship at Gresham College in 1890 ( Magnello, 1996: 47). The latter appointment connected Pearson with the zoologist Walter Frank Raphael Weldon. Together, they developed research applying quantitative methods to biological questions.

Weldon also introduced Pearson to Galton. In 1892, Petrie became next-door neighbours with Pearson ( Drower, 1995: 222, 260, 339). Around this time Pearson, possibly influenced by Galton’s work, was searching for appropriate material for a craniometric study. In 1894, Petrie agreed to set aside skulls from his forthcoming excavation at Naqada in Egypt, and in the following year, the skeletal remains of over 400 individuals were sent to Pearson in London ( Clever, 2020: 92–93; Fawcett & Lee, 1902). The craniometric results of this study were published in 1902 in the newly-established *Biometrika,* ^4^ and though only a brief mention is given of the sex estimation of the skulls ( Fawcett & Lee, 1902: 411), there is a detailed description of the Frankfurt protocol for measurements ( Fawcett & Lee, 1902: 413–419). This publication was noted both nationally and internationally, and ‘cemented the importance of skulls in biometric work’ ( Challis, 2016: 6).

Though the UCL Biometric Laboratory, led by Pearson, was more statistically oriented than The Francis Galton (later National) Eugenics Laboratory at University College, both Pearson and Galton shared an interest in defining ‘racial’ or 'population' differences ( Challis, 2016; Porter, 2004: 280) —terms that were often used interchangeably by contemporary scientists, including *Biometrika* authors. Pearson statistically defined Galton’s Law of Ancestral Heredity, leading to the development of multiple regression analysis ( Pearson, 1896). Pearson became the director of both laboratories in 1911 and continued to conduct craniometric research ( Pearson, 1938: 187).

Petrie sent numerous skulls and other skeletal remains to Pearson over the decades, including human remains from Gizeh ( Petrie, 1907b: 29), Lahun ( Petrie *et al.*, 1923: 22), and Abydos ( Petrie *et al.*, 1925: 8, 9). He continued to do so after moving his projects to Palestine, up until around the time of Pearson’s retirement in 1933. ^5^ These joined human remains from other sources; by the time of Pearson’s death in April 1936, his collection as a whole was said to contain around 7000 skulls ( Pearson, 1938: 216).

### 6.3 Subsequent research and publication

The osteological remains that Petrie sent to the UCL Biometric Laboratory from Egypt received considerable attention from contemporary researchers, leading to a number of publications (e.g., Hoadley & Pearson, 1929; Martin, 1936; Morant, 1925; Morant, 1935; Morant *et al.*, 1936; Stoessiger, 1927; Woo, 1930). However, the Palestinian skulls he donated seem conspicuously absent from the pages of *Biometrika* and elsewhere. This anomaly piqued our interest and lead to a detailed investigation of archival sources. Had there been research activity on this collection? And if so, why did it not lead to any published reports?

The archives proved informative. In a letter to Karl Pearson discussing skulls from his excavations at Lachish, Leslie Starkey makes passing reference to the ‘amount of work Dr. Morant has put in on the skulls of the Wadi Ghuzzeh district’ ( Starkey, 1933). Morant was a student of Karl Pearson, who went on to become one of his Biometric Lab’s main researchers ( Barkan, 1991: 158–162; Clever, 2020: 33, 176). All three of Petrie’s Palestinian sites — ʿAjjul, Fara and Jemmeh — were located along the Wadi Ghazzeh, and so this must be the material Starkey is referring to here. However, Morant’s involvement may have been managerial, rather than analytical, as he later commented that none of this Petrie material had been measured (which was not strictly true, as will be seen below), and he showed little knowledge of the archaeological sources from whence it had come ( Trevor, 1946b: 2). While Morant’s research interests were primarily focused on anthropometrics ( Clever, 2020: 180), none of his published work appears to have involved the Palestinian skulls in the Pearson lab.

In 1933, Pearson agreed that his Biometric Laboratory would host work on a large series of skulls from Starkey’s excavations at Lachish ( Pearson, 1933). These were assigned to former Petrie excavator D.L. Risdon, who was given a special grant from the laboratory to conduct the research; his results were published in an issue of *Biometrika* ( Risdon, 1937; Risdon, 1939). From Risdon’s unpublished research notes, which we discovered in the archives of the Natural History Museum, it is clear that during this period Risdon also had access to the skeletal remains Petrie had collected in southern Palestine, both ancient and modern, which were seen as a valuable source of comparative data. He worked on this material as intensively as he had on the Lachish assemblage, applying to it the various methods of statistical analysis favoured by Pearson, and most probably introduced to him by Morant. He even appears to have drafted an 11-page report on the assemblage, which was never published ( Risdon, n.d.). Despite this, Risdon himself downplayed his efforts on this group, later telling Geoffrey Morant and Olga Tufnell that the data he had collected from it ‘should be ignored’ ( Morant, 1946; Tufnell, 1946a). The Second World War interrupted his research — Risdon went to work for the Air Ministry — and it was never resumed. Risdon’s working notes and calculations were eventually deposited with the Natural History Museum, including the data he had collected on Petrie’s Palestinian skulls, the Lachish series, and others from ancient sites in Egypt ( Natural History Museum, n.d.).

## 7. The Duckworth Laboratory

### 7.1 How Petrie’s material came to be sent to Cambridge

On hearing of his retirement, Petrie wrote to Pearson, requesting that the skeletal human remains that he had sent him over the years be retained in the laboratory, rather than being ‘regarded as general anatomical material;’ its value, as he put it, was that this group ‘especially provided for the mathematical treatment which cannot be applied to small quantities’ ( Petrie, 1932b). This underscores the fact that Petrie viewed this assemblage as a statistical series rather than as items of individual interest. Both Pearson, and his protégé Morant, who remained working at University College, would certainly have supported this view, and there is no hint at this time that the material might be disposed of. However, the fate of the assemblage was not in their control, and as it turned out, the Biometric Laboratory and its skull collections was to fall victim to departmental restructuring and a change in direction taken by Pearson’s successors.

After Pearson’s retirement in 1933, the department was split into the Eugenics Department (later Human Genetics), and the Department of Statistics. Pearson was succeeded by Ronald Fisher as the Galton Professor of Eugenics. While Petrie did exchange ideas with Fisher (e.g.: Fisher, 1929), he never collaborated closely with him, as he had with both Galton and Pearson. This was most probably because Fisher preferred to work with living populations rather than ancient remains ( Clever, 2020: 148). As an adversary of Pearson who was openly critical of his craniometric methods ( Clever, 2020: 146–148; Fisher, 1936), this appointment put the future of Pearson’s skull collection in doubt. On Karl Pearson’s death, the collection became the responsibility of his son, E.S. Pearson, and was clearly considered to be a private assemblage, rather than a college asset. Meanwhile, Morant was allowed to continue his craniometric research on the collection under the new arrangements, with E.S. Pearson’s support, but felt Fisher was being ‘covertly hostile’ to his work, and that his career prospects had stalled ( Clever, 2020: 184).

Fisher left the Galton Chair in 1944 and was succeeded in the post by Lionel Penrose in the following year. After the atrocities carried out in the name of eugenic research and theory during World War II came to light, Penrose led a shift in his department towards genetics, as part of a wider movement away from eugenics within his discipline ( Valles, 2012). In the same year, Morant decided to leave University College ( Morant, 1945), and on the back of that, E.S. Pearson agreed to transfer his father’s collection — now being stored in a college basement — to Cambridge University, where it became part of the newly-founded Duckworth Laboratory ( Hutton, 1945; Lahr, 2011; Pearson, 1945; University of Cambridge, 1947: 4), named after the anatomist Wynfrid Lawrence Henry Duckworth (1870–1956).

Geoffrey Morant was given the task of packing up the ‘Pearson Collection’ in London, with E.S. Pearson presenting his father’s material — including Petrie’s many contributions from both Palestine and Egypt — to the Duckworth Laboratory in late 1945. The collection was now said to comprise some 10,000 crania and other bones ( University of Cambridge, 1947: 5). The collection was accompanied by a summary list ( Trevor, 1946b). The author of this list, Morant, did not appear to be well informed of the sources of the material ( Morant, 1946); he erroneously attributed the skeletal human remains from Petrie’s fieldwork in Palestine to the ‘Wellcome-Marston Expedition’ (the official sponsors of the Lachish excavation), despite correctly naming two of the three sites from which they had derived ( Trevor, 1946b: 2). The Lachish skulls were not actually included in this transfer, as they had already been sent to the British Museum of Natural History ( Fraser, 1940).

After arriving at the Duckworth, the Palestinian material maintained its profile for some years, largely due to the intervention of Olga Tufnell, who corresponded with the Duckworth’s new director, Jack Trevor in the hope of seeing it studied and published. Under her encouragement, Trevor arranged a grant that enabled one of his students, Ian Cunnison, to measure the Petrie skulls ( Trevor, 1946a). Cunnison does not appear to have had access to the unpublished data previously collected by Risdon. Meanwhile Tufnell, who had taken on the responsibility for seeing the Lachish results published, arranged for the Lachish skulls to be loaned to the Duckworth so they could be included in this work ( Tufnell, 1946b). Between them, they made arrangements for further analysis and publication, the idea being that the focus would be on the Lachish skulls, with other modern and ancient Palestinian skulls being brought in as comparanda ( Trevor, 1948; Tufnell, 1948). This plan was derailed, however, by a misunderstanding with the person hired to do the job, Madeleine Giles (later Smith), who only analysed the Lachish material, much to everyone’s dismay ( Giles, 1950; Tufnell, 1950). Her reports were published in *Lachish III* and *IV* ( Giles, 1953; Giles, 1958), and while hopes were expressed that the Petrie material could form the subject of further research ( Tufnell, 1951), including an attempt to persuade Jack Trevor to write a report on some of the skulls from the courtyard cemetery at ‘Ajjul ( Tufnell, 1960; Tufnell, 1961), these plans came to nothing.

The lack of visibility of the curated human remains from Petrie’s Palestinian sites was therefore not down to a lack of interest or appreciation of its potential, but a result of bad timing and logistical issues. Pearson, a supporter of this work, died in 1936. The two men largely responsible for collecting the material, both of whom showed a strong interest in craniometric studies, passed away not long afterwards: Starkey in 1938, and Flinders Petrie in 1942. Risdon, who was clearly interested in the Palestinian skulls, did not continue his osteological work after the war, while his mentor in the Biometric Laboratory, Geoffrey Morant, similarly left physical anthropology around the same time ( Clever, 2020: 217; Morant, 1945). Thereafter, the collection’s new custodian at the Duckworth, Jack Trevor struggled to find suitable researchers to work on the Petrie material, despite managing to secure funding for the task. And once Tufnell, who was extremely vocal in support of the value of this assemblage, had fulfilled her Lachish obligations, she moved on to other projects. So the Petrie Palestinian material was set aside, and, it would seem, eventually forgotten. In contrast, the skulls Petrie had collected in Egypt continued to attract research attention (e.g., Johnson & Lovell, 1994; Leek, 1972; Miller, 2008), even as the knowledge of how these had come to be in Cambridge was being gradually lost. ^6^

### 7.2 The Petrie Palestinian material in the Duckworth Collections

***7.2.1 Scope and marking systems***

The Duckworth Laboratory curates approximately 18,000 individuals and thus forms one of the largest collections in the world ( University of Cambridge, n.d.). Within this, over 100 boxes are attributed to human skeletal remains from Petrie’s excavations in Palestine. These were originally inventoried in a handwritten, loose-leaf catalogue, created at some point after the material arrived in Cambridge and now housed in the Duckworth archive. This records the ‘old’ catalogue number, which was assigned in Pearson’s laboratory in the 1930s, alongside its ‘new’ Duckworth number. This information was subsequently incorporated into the Duckworth Laboratory database. A summary of this material is outlined in Table 5.

**Table 5.** Summary of the Petrie Palestinian material in the Duckworth Collection. Collection dates reflect the fact that most field seasons ran from November or December through to April the following year.

Series	Site	Collection date	Pearson No.	Duckworth Laboratory No.
Modern Arab	Tell Fara	1927–28	F1–F61	As.66.0.1–61
	Tell Fara	1928–29	F/A1–A9	As.66.0.62–70
	Tell Fara	1929–30	[AR] 1–4	As.66.0.71–74
	Tell Fara or Tell el-ʿAjjul	1930–31	II	As.66.0.75
	Tell Fara (disassociated mandibles)	1927–28?	F14 F43 F52 F62 F63	As.66.0.76 As.66.0.77 As.66.0.78 As.66.0.79 As.66.0.80
Medieval Arab	Tell Jemmeh	1927	G1–G4	As.66.1.1–4
Ancient	Tell Fara, Tell el-ʿAjjul	1927–33	P1–P42	As.66.4.1–42
	Tell el-ʿAjjul (disassociated mandibles)	1930–31 1933–34	P P P	As.66.4.43 As.66.4.44 As.66.4.45
	Tell Fara (disassociated long bones)	1929–30	None	None

The ‘old’ numbers are written on individual skulls or bones in large black ink markings, sometimes duplicated in blue pencil. As the style of these markings does not match what Petrie’s team were doing in the field, it seems likely that these were assigned in Pearson’s Biometric Laboratory at UCL as a way of managing the large numbers of skulls they were acquiring. From the way these Pearson numbers were allocated, it is possible to deduce that the Petrie material originally arrived in the UCL laboratory in groups at the end of each field season, where items were divided into series based on their date: modern Arab (F-prefix, F/A-prefix, and no prefix), medieval Arab (G-prefix) or ancient (P-prefix). Some prefixes chosen seem to represent site codes (‘G’ = Gerar, aka Tell Jemmeh; F = Fara), or a broader classification (P = Palestine, grouping material from Fara and ʿAjjul; Trevor, 1946a), but the modern Arab numbering system lacked consistency from year to year.

The Duckworth Laboratory subsequently assigned numbers from its own cataloguing system to each individual. These maintained the original Pearson series groupings and were coded to record continent (As = Asia), country (66 = Palestine) and period (0 = modern or unidentified, 1 = medieval, 4 = Bronze Age). The numbers were also physically marked on each item.

As Table 5 demonstrates, both the modern and ancient material included a number of disassociated mandibles and long bones. It is not clear if these lost their association with specific crania in the field, or after arriving at University College. This problem was not limited to mandibles in Petrie’s Palestinian assemblages. E.S. Martin, who published a study on Petrie’s Gizeh mandibles in 1936, noted that:

Of the mandibles, all that is known is that they probably form a selection from the original 1800 mandibles belonging to the 1800 crania, but it is not known to which cranium any individual mandible belongs […] My impression is that more mandibles than crania were brought from Gizeh, possibly many too fragmentary to be preserved. Dr Morant found only 600, but at my pressing request, he has recently further searched the stores and found another nearly complete 200. The numbering is peculiar, and seems to indicate that farther mandibles must have been found originally. This somewhat weakens the argument that the mandibles belonged in bulk to the 1800 crania.
  ( Martin, 1936: 149)

This suggests that by the 1930s there was already considerable mixing and loss of curatorial integrity within the Pearson Collection, arguing against a later suggestion that this damage and disassociation might have occurred during the Second World War ( Leek, 1972: 290).

***7.2.2 The ‘Modern Arab’ series of human remains from Tell Fara***

The human remains found at the Duckworth Laboratory make it clear that Petrie did not confine himself to excavating ancient burials. Individuals marked with F, F/A, [AR] and II-prefix series numbers were described in the records as ‘modern Arab’, collected at Tell Fara between late 1927 and 1930. An additional skull collected in 1930–1931, and given a ‘II’-prefix could have come from either Fara or ʿAjjul, as both sites were being worked at the same time. In contrast to the P-series, none of the 81 skulls or skeletal remains identified as modern or medieval Arab were marked with tomb references, pointing to different treatment of human remains depending on their perceived antiquity.

***7.2.3 The ‘Medieval’ human remains from Tell Jemmeh***

The whereabouts of most of the Tell Jemmeh skeletal human remains is unknown, but the Duckworth Collection includes four skulls attributed to Petrie’s excavations at the site, said to be ‘Medieval Arab,’ and brought back by Petrie at the conclusion of his Jemmeh field season in 1927 ( Table 5: G1–4). This attribution might suggest they derived from the burials on the tell mentioned earlier, and indeed, this is supported by a letter Olga Tufnell wrote to Jack Trevor at the Duckworth Laboratory which cross-references Petrie’s mention of these ( Petrie, 1928a: 25; Tufnell, 1946a).

Tufnell was unable to offer a more specific date for this group of human remains ( Tufnell, 1946c: 2). However the term ‘medieval’ should probably be taken with a grain of salt, as in a later, unsigned letter sent to Jack Trevor about this material, the author states:

“Medieval”, by the way, is a deliberately vague term applied to burials between the 16th and 19th centuries A.D., to avoid giving offence when these graves have been disturbed.
  ( Anon, 1950).

The lack of specific context and field documentation makes it impossible to contextualise the Jemmeh skeletal human remains further. All the skulls in this group had been waxed.

***7.2.4 The Ancient human remains from Tell Fara and Tell el-ʿAjjul***

Material in the P-series derived from burials excavated at Tell Fara and Tell el-ʿAjjul. These were properly documented in the field, using the systems described earlier in this article, and the majority were labelled with their tomb numbers. There are only four exceptions: P19, P21, P24 and P25, which lack context markings. Letters in the British Museum Lachish archives suggests that these individuals had already been disassociated from their provenance details by the 1940s, and it is unlikely that this data can now be recovered ( Cunnison, 1946; Tufnell, 1946c: 2).

Within the P-series, there is no obvious logic to how the material was ordered. It does not appear to be grouped by site, context, period, age or sex — metric sex estimation often being the focus of Pearson’s articles. Perhaps skulls were just numbered as they came to hand when the material was catalogued at University College. While it seems logical to assume that some sort of handlist was kept, in order to prevent duplicate numbers being assigned, this has not yet been located. It is worth noting that when offering the collection to Cambridge in 1945, E.S. Pearson admitted that there was ‘no detailed list’ available ( Pearson, 1945). Trevor was later given a summary of the Palestinian material, put together specifically for the transfer by Geoffrey Morant ( Trevor, 1946b). The ancient human remains attributed to Tell Fara and Tell el-‘Ajjul are summarised in Supplementary Tables 1–2 ( Refer extended data).

### 7.3 Osteological analysis of selected Petrie material in the Duckworth Collection

In 2018 Nina Maaranen visited the Duckworth Laboratory to examine 41 P-series individuals from Tell el-ʿAjjul and Tell Fara as part of the Hyksos Enigma research project ( Maaranen, 2020). The initial aim was to record teeth for a biodistance analysis to help understand migration and mobility in the eastern Mediterranean during the Middle Bronze Age ( *c.* 2000–1550 BCE). However, this was also an opportunity to investigate other aspects of the assemblage, including age, sex, and other notable features. The results of this analysis are presented below and in Supplementary Tables 1–2 ( Refer extended data).

***7.3.1 Age estimations***

As discussed earlier, specific age estimates were provided only rarely in Petrie’s field records, appearing on just 1.6% of extant tomb cards ( Table 3). However, broader terms such as ‘adult,’ ‘young adult,’ and ‘child’ appear both in the tomb cards and published text, albeit lacking explanation as to their scope or the methods used. Slightly more detail was provided in a report written by Karl Pearson on selected cremation burials from Fara Cemetery 200, published in *Beth Pelet I* ( Petrie, 1930a: 13), which also comments on the fragmentary state of the Tell Fara material. The short description does give some clues to the features used to assess age, such as cranial suture closure and tooth wear.

Re-examination of the provenanced P-series skeletal remains in the Duckworth Laboratory revealed that most individuals were either adults or on the cusp of adulthood Supplementary Tables 1–2 ( Refer extended data). ^7^ Because this sample was primarily made up of skulls, it has only been possible to give numerical age estimations to late teens who possessed still-forming second and third molars. Skulls which show eruption of the third molar have been identified simply as ‘adult,’ though in some cases a distinction between young or old adults has been made on the basis of tooth wear and suture closure. It should be noted that tooth wear patterns alter according to diet, with gritty food leading to stronger attrition, while suture closure can give a large age range of 40 years (e.g., from 25 to 65). The bias towards adults suggests that Petrie and his staff had deliberately excluded young sub-adults when sending material to England for further study. This is not surprising given the interest at that time in craniometric studies and ancestry, as this type of research requires adult crania; it would appear that Lachish excavators also gave less attention to the skulls of children for this reason ( Risdon, 1939: 103). Risdon’s unpublished manuscript does mention immature individuals, which likely refers to the adolescents/young adults found in the Duckworth Collections.

***7.3.2 Sex estimations***

Sex estimation is an equally important component of skeletal analysis, so it is not surprising that Petrie’s team appears to have attempted some sex identifications in the field, with 9.2% of the tomb cards providing data on this ( Table 3). While Petrie used this information when describing the cemetery material from Tell Fara and Tell el-ʿAjjul, he did not discuss how the data had been obtained, who collected it, what criteria were applied, and whether sexing was done of skeletal human remains *in situ,* or only after the bones had been removed to the dighouse. To understand practical aspects of estimating sex on a Petrie dig, therefore, other sources of evidence are required.

Back in the 1890s, Petrie appears to have been using both skull and pelvis to identify sex ( 1897: 25). Similarly, we know that one Murray-trained Egyptologist, Guy Brunton, was sexing skeletal human remains at Matmar in 1930 by examining both skull and pelvis *in situ* ( Young, 2021: 167); like Petrie, he commented on the importance of the pelvis in sex identifications ( Brunton, 1927: 5). Starkey would no doubt have been influenced by his time working for both men at Qau in the early 1920s, where he recorded many burials, and we can expect him to have used their methods, which could then have been passed on to his other colleagues in the field. However, we cannot assume that the same level of accuracy was achieved by field staff with less experience or training.

Outside of the field, Pearson, Morant, and others accessing the materials in the Biometric Laboratory at University College frequently incorporated sex estimations in their research. While their authors do not explicitly state their methods for achieving this, several referenced the work of George Murray Humphry, professor of physiology and anatomy at the University of Cambridge (e.g., Pearson & Davin, 1921; Woo & Pearson, 1927). His book *A Treatise on the Human Skeleton* ( 1858) provided a description of sex-based differences, so it is possible that Humphry’s observations, or others that followed the same vein, were used to estimate sex in Pearson’s collections as well. Though many of Humphry’s observations are still widely accepted, there is now a greater understanding of the subjectivity and population-specificity of sexual dimorphism ( Walker, 2008). As D.L. Risdon was trained by Morant, we can assume that he adopted the Biometric Laboratory’s methods for his sex identifications of Petrie’s skulls from his Palestinian sites when researching this material, the results of which sadly remained unpublished ( Natural History Museum, n.d.; Risdon, n.d.).

It would appear that there were common physical criteria being used to estimate biological sex, however these were not the only criteria being used. A few of Petrie’s ʿAjjul tomb cards include explanatory notes that indicate either physical or associative evidence could be employed when determining the sex of a burial. So while at times we are presented with anatomical justification for the suggested sex (e.g., ʿAjjul Tomb 2028: ‘from stature and size of bones a male’), on other occasions we see sex being assigned by associated finds (ʿAjjul Tomb 2065: ‘woman from beads at neck’). A clear gender-bias was in operation here, with beads being flagged as ‘female’ artefacts (e.g., ʿAjjul Tombs 2065, 2080, 2134 — see Figure 2), and objects such as spears and daggers as ‘male’ (e.g., ʿAjjul Tomb 2008). Likewise, in Egypt, hair styles were also sometimes utilised as indicators of sex of ancient burials ( Morant, 1935: 298–299), a practice that currently would only be used to explore gender identities. This may be why a comparison made of the sex estimations performed on a series of skulls from Petrie’s excavations at Badari showed a low level of agreement ( Morant, 1935: 299). These methods bring the reliability of the sex data being recorded into question. Still, it should be noted that field staff had an advantage over lab workers when determining sex, as they had access to full skeletal remains *in situ* rather than just the skull. The lower reliability and subjectivity of sexually dimorphic features of the skull could explain why even subsequent lab-based analyses were not in complete agreement ( Morant, 1935: 298; see also the discussion below).

Because of the uncertain sources of evidence used, a reappraisal of the surviving skeletal remains of Petrie’s Palestinian sites seemed appropriate. During her visit to the Duckworth Laboratory, Maaranen conducted sex estimations of 41 ancient individuals from identifiable contexts. ^8^ The results are presented in Supplementary Tables 1 and 2. These results should be considered tentative, as sex estimations based on skulls alone will not be as reliable as estimations based on the entire individual, due to age- and population-based variation. For instance, Zakrzewski (2007) has reported on the inflated size of the mastoid processes on ancient Egyptian females. Whether this holds true for Levantine populations as well has not been investigated.

It was hoped that this reappraisal could also be used to test the accuracy of the original field identifications of the skeletal human remains, as recorded on the tomb cards. Unfortunately, of the material preserved in the Duckworth, only two individuals appear to have been sexed in the field. The first of these was a skull from ʿAjjul Eastern Cemetery Tomb 412 ( Petrie, 1931a: pl. LXI). The tomb card identified the two interments in the tomb as female; whereas a single skull from this tomb found in the Duckworth Collection has been identified as an adult, possibly male (As.66.4.33). Another seeming mis-match occurs in the case of ʿAjjul Tomb 456. The tomb card identified the remains as two adult females and a child, whereas a single mandible in the Duckworth is attributed to this tomb, but also identified as an adult, possibly male (As.66.4.45). Neither case is conclusive, however, and it should be noted that the excavators appear to have had access to the complete skeletal human remains for one of the burials in Eastern Cemetery Tomb 412, and all three burials in Tomb 456, judging by the sketches on the back of their respective tomb cards, so may have been able to utilise pelvic bones when establishing sex.

***7.3.3 From ‘race’ to modern biodistance analysis***

Biodistance analysis is conducted using a variety of statistical tools, and though some statistical elements had already been incorporated into the metric studies by the 19th century (e.g., Broca, 1863; Morton, 1844), the foundation for currently employed methods was laid in the beginning of the 20th century (e.g., Campbell, 1925; Hrdlička, 1927; Krogman, 1927; Shaw, 1931). Karl Pearson (1926) himself created the ‘Correlation of Racial Likeness’ (CRL), a technique that compares groups based on measurements. The method was employed by those working in Pearson’s Biometrical Laboratory (e.g., Batrawi & Morant, 1947; Morant, 1925; Morant *et al.*, 1936; Risdon, 1939; Stoessiger, 1927), despite being proven to fail to account for inter-variable correlation ( Mahalanobis, 1936: 418); it was not adopted more widely.

In the past four decades, the field has developed immensely due to the ongoing improvement of computing power, including methods that allow investigators to conduct analyses even on incomplete and fragmentary data. Concurrently, anthropological research has become increasingly aware of the problematic studies of so-called ‘race’ that were common in Petrie’s day, and consequently moved to focus on questions of geographical variation, local (intra-site) population affinities between people, and the dynamic relationship between biology and the environment. In this context, the term ‘race’ is understood as a purely cultural construct, with biological consequences that take on an appearance within the framework of a population ( Armelagos & Van Gerven, 2003).

Data for the current project was collected from Tell el-ʿAjjul and Tell Fara burials ranging in date from the Middle Bronze Age through to the Iron Age periods, using the Arizona Dental Anthropology System (ASUDAS) of data collection, as this assemblage was accessed with particular research questions in mind. For discussion of this method and its previous application to Egyptian and Near Eastern materials, see Maaranen, Zakrzewski and Schutkowski (2022). Table 6 outlines the list of traits recorded.

**Table 6.** Dental non-metric traits recorded from the Tell Fara and Tell el-ʿAjjul individuals. Data was collected using the Arizona Dental Anthropology System (ASUDAS). Column ‘tooth’ indicates the primary tooth the trait is recorded from; U = upper, L = lower, I = incisor, C = canine P = premolar and M = molar. For instance, UI indicates upper (U) first incisor (I1).

Trait	Tooth	Recording Scale	Abbreviation
Winging	UI1	Grade	W
Labial curvature	UI1	Grade	LC
Palatine torus		Grade	PT
Midline diastema	UI1	Present/absent	MiD
Shovelling	UI1	Grade	S
Double-shovelling	UI1	Grade	DS
Interruption groove	UI2	Present/absent	IG
Tuberculum dentale	UI2	Grade	TD
Pegged or reduced incisor	UI2	Grade	UI2V
Mesial accessory ridge	UC	Grade	MAR
Distal accessory ridge	UC	Grade	DAR
Premolar accessory ridges	UP	Grade	PAR
Accessory cusps	UP	Present/absent	AC
Upper premolar root number	UP	Count	RN UP
Metacone size	UM	Grade	M
Hypocone size	UM	Grade	H
Cusp 5	UM	Grade	C5 UM
Carabelli cusp	UM	Grade	CC
Parastyle	UM	Grade	PA
Enamel extensions	UM	Grade	EE
Upper molar root number	UM	Count	RN UM
Reduced or agenesis	M3	Present/absent	M3.red.abs
Odotome	P	Present/absent	O
Mandibular torus		Grade	MT
Rocker jaw		Grade	RJ
Lower canine root number	LC	Count	RN LC
Tome's root	LP1	Grade	TR
Lower premolar lingual cusp number	LP	Count	CN
Deflecting wrinkle	LM1	Grade	DW
Mesial and distal trigonid crest	LM1	Grade	MTDC
Anterior fovea	LM	Grade	AF
Groove pattern	LM	x/y/+	GP
Cusp 5	LM	Present/absent	C5 LM
Cusp 6 size	LM	Grade	C6
Cusp 7 size	LM	Grade	C7
Protostylid	LM	Grade	PR
Lower molar root number	LM	Count	RN LM
Torsomolar angle	LM3	Present/absent	TA

**Figure 9.**
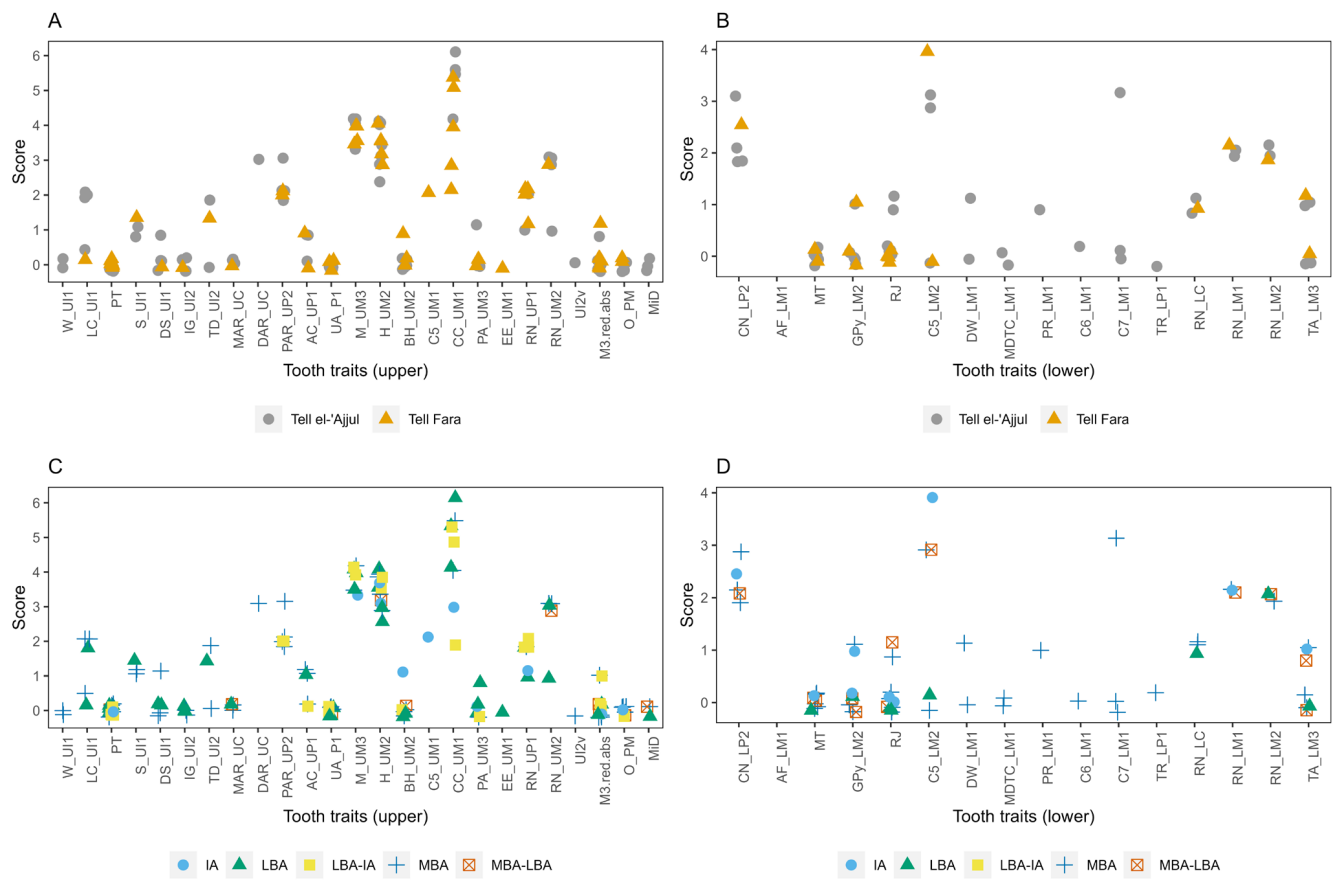
Dental non-metric trait scores by site ( **9A** and **B**) and by period ( **9C** and **D**). Sites were pooled for the trait scores by period. The first part of the abbreviation refers to the dental trait, followed by tooth designation after the underscore (e.g. H_UM2 = hypocone size (H) on the upper (U) second molar (M2)). See Table 6 for a list of abbreviations used.

Dentition in the Tell Fara and ʿAjjul collections was largely missing, a common occurrence for early modern skull collections. Risdon commented on the poor condition of the Palestinian skeletal human remains in the Biometric Laboratory ( Risdon, n.d.: 3), which Pearson appears to have attributed to poor packing ( Pearson, 1933: 2). The fragmentary and incomplete nature of the Tell el-ʿAjjul and Tell Fara material limited the biodistance analysis, however some general, if tentative, observations can be offered. The plotted individual scores in Figure 9 exemplify the scarcity of available data. The raw data has been included in Supplementary Material. Figures 9A and 9B suggest a general overlap between dental trait scores from Fara and ʿAjjul. There is a wider range of UM1 Carabelli cusp values at Fara, and the scores forming the lower range of the scores seem to date to the Late Bronze–Iron Age transition and Iron Age period ( Figures 9C and 9D). Other traits that stand out, this time forming the upper ranges, are the UM2 bifurcated hypocone and LM2 cusp 5 size, also from the Iron Age Fara sample. Though these could point to slight differences in population structure that begin to take place in the Iron Age (e.g., newcomers), it could also be an effect of the small sample size, as most of the Iron Age trait scores identified in this assemblage do overlap with other time periods.

To explore how the data from Tell el-ʿAjjul and Tell Fara fit into wider Southern Levantine assemblages, an exploratory analysis was conducted using Bronze Age data from Jericho and Pella. The chosen method, Gower Distance Analysis, creates a distance value for each individual, rather than group. This was chosen because while the most commonly used group analysis method, Mean Measure of Divergence (MMD), is robust and can accommodate missing data, the statistical analysis it produces can become biased by too small sample sizes (i.e., n<10). Gower Distance Analysis has the benefit of comparing individuals rather than groups, however this also has its drawbacks. While Gower Analysis can accommodate missing data, it is in Maaranen’s experience that the dataset still requires around 70% completeness. A detailed description of the methods and pre-analysis checks is available in Maaranen, Zakrzwewki and Schutkowski (2022) and Stantis *et al.* (2021). Because of the incomplete dentition at Tell el-ʿAjjul and particularly Tell Fara, there were only a few individuals that provided enough data for a multi-site comparison. Hence, the descriptive and tentative nature of these observations must be underlined and taken into account for any future work.

A total of 24 individuals were suitable for the analysis: one from Tell Fara, five from Tell el-ʿAjjul, three from Pella and 15 from Jericho ( Supplementary Material). Fourteen dental non-metric traits were selected across the four sites: upper first molar Carabelli cusp (CC_UM1) and enamel extension (EE_UM1); upper second molar metacone (M_UM2), hypocone (H_UM2), bifurcated hypocone (BH_UM2) and cusp 5 (C5_UM2); third molar congenital absence (M3V); second lower premolar cusp number (CN_LP2); first lower molar groove pattern ‘X’ (GPxLM1), cusp 5 size (C5_LM1) and cusp 7 (C7_LM1); second lower molar anterior fovea (AF_LM2), groove pattern ‘Y’ (GPyLM2) and protostylid (PR_LM2).

**Figure 10.**
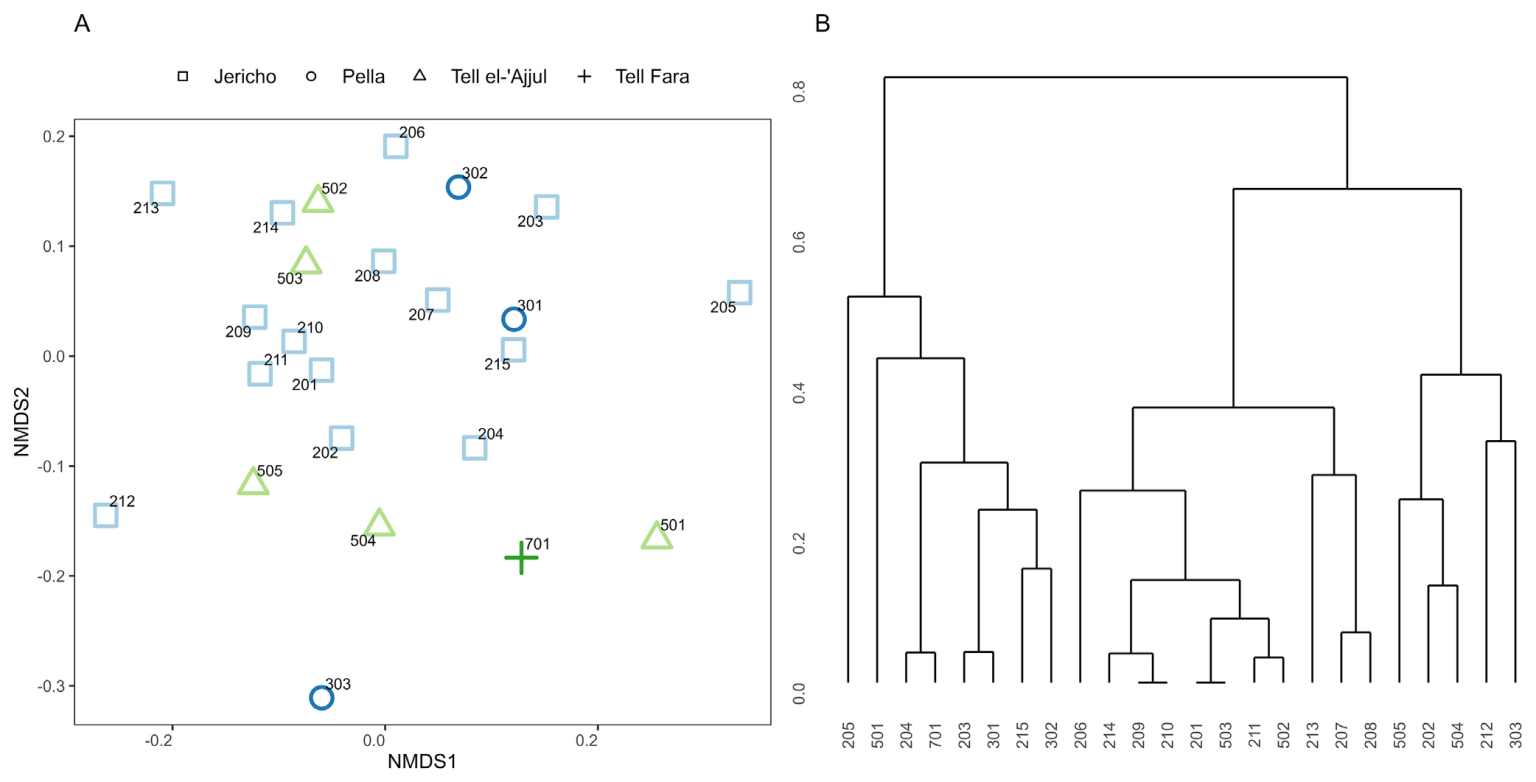
NMDS plot ( **A**) and hierarchical cluster analysis dendrogram ( **B**), showing site dispersal and overlap between sites. Arbitrary ID numbers have been assigned to simplify the graphs. The 200-series refer to Jericho, the 300-series to Pella, the 500-series to Tell el-ʿAjjul, while 701 is Tell Fara. For ID numbers from Tell el-ʿAjjul and Tell Fara, see Supplementary Tables 1 and 2; a complete list is available in the extended data.

A non-metric multidimensional scaling (NMDS) plot was produced from the distance matrix ( Figure 10A), indicating an overlap of biological similarity between sites; this was also evident in the hierarchical cluster analysis ( Figure 10B).

As Jericho has provided the largest subset of data, this also offers the best comparative evidence. Jericho individuals seem to form a loose cluster, surrounded by individuals from the other sites: three out of five from Tell el-ʿAjjul (‘High Tomb’ at level 990, Well 2 in Cemetery 100, and Tomb 626 from room AV), one from Tell Fara (Tomb 960, Body B) and one from Pella (XXVIIIA 8.10, F.10, Burial A). The samples focus on the Middle Bronze Age ( *c.* 2000–1550 BCE), a period of increasing regional trade and interconnectivity ( Ilan, 1995) which changed population structures at other contemporary sites such as Tell el-Dab’a ( Maaranen *et al.*, 2022).

## 8. Discussion

This article was inspired by our rediscovery of the ‘lost’ human bone from Petrie’s excavations in Southern Palestine, and recognition of its untapped research potential. In trying to contextualise this material, we soon came to realise how little information was available in the published record — and yet how much could be learned from a close study of archival sources. It was felt that these small clues were worth pursuing, as they add to an understanding of how skeletal human remains were excavated, recorded, and curated — processes which have helped shape the Duckworth assemblage in its current form. Moreover, on a wider scale, understanding the intellectual climate and background to the formation of historical collections of osteological materials is important, as it allows us to identify potential bias in sampling, research and analysis — and the way in which these might affect modern attempts to use the material objectively.

Throughout his career, Petrie viewed the human remains he encountered in his projects as a means to an end. Like most of his contemporaries, there is little indication that he had any concerns about exhuming, studying, or discarding the dead — and, with the rare exception of one documented reburial at Abydos ( Petrie *et al.*, 1925: 9), done for unstated reasons — human remains were foremost treated as scientific ‘specimens.’ While Petrie certainly recorded anatomical information in the field about age and sex, which he fed into his excavation reports, it appears that he retained human remains primarily for their anthropometric value. The curated content of the Palestinian collection at the Duckworth seems to reflect this agenda in the exclusion of subadults — whose incomplete cranial development would make them unsuitable for craniometric analyses — and the somewhat equal distribution of males and females, which would be suited to sex-based comparative studies. Another obvious selection criterion was surely the preservation and completeness of the skull, which the excavators attempted to maintain post-excavation by coating with paraffin wax. Teeth were seldom of interest in this type of research, which may explain their low frequency in the Duckworth assemblage.

One of the most problematic aspects of Petrie’s study of human remains was the way that these were further used to promote his personal views on inherited mental qualities and eugenics ( Challis, 2013; Challis, 2016; Gange, 2013: 276–280; Petrie, 1907a: 4; Sheppard, 2010). For example, in his book, *Janus in Modern Life*, Petrie ( 1907a: 89) promoted, among other things, the idea of human sterilisations to ‘improve stock.’ *Janus* was published in 1907, the same year the British Eugenics Education Society was founded with Sir Francis Galton as its honorary president ( English Heritage, n.d.). The views expressed in works like this do not sit comfortably against the image presented by Petrie’s colleagues, friends and biographers of a great ‘Father of Egyptology’, who did so much to advance the progress of archaeological research. It is a reminder how much is often glossed over when writing the histories of the development of intellectual thought.

We cannot tell to what extent Petrie or Pearson’s ideas on race and eugenics were shared by the people working for them. The terminology used by researchers at this time is not completely clear-cut. In the journal *Biometrika*, ‘race’ seems to have been used as a synonym for population, as it was used to divide people by sites (e.g., Batrawi & Morant, 1947; Fawcett & Lee, 1902; Morant, 1935; K. Pearson, 1926; Risdon, 1939; Woo, 1930). Many anthropologists, biologists and statisticians helped promote and drive eugenic research forward, with the use of race as a biological classification. This has been described as ‘the original sin of anthropology’ ( Levi-Strauss, 1952: 1). Despite the criticism and opposition of some contemporary anthropologists, such as Franz Boas (1916), the rising political climate of Europe and Northern America favoured the growing eugenics movement. As a result, many Western countries, such as USA and Germany, began performing sterilisations based on ‘mental disabilities’ ( Reilly, 2015). In the UK, despite several attempts, bills allowing forced sterilisation were never passed.

Petrie’s links with the eugenics movement are not the only ethical issue raised by his treatment of the human remains he encountered. One unexpected outcome of our investigations was the realisation of how many skulls had been collected from historic and modern burials in Palestine, despite their almost complete absence from the official record. There is no evidence that Petrie’s team assigned tomb numbers to the post-medieval graves they discovered; no records seem to have been made of their contents, and certainly no plans or descriptions were ever published. When one examines the correspondence and personal diaries of Petrie’s field staff, it becomes clear also that ‘modern’ human remains were viewed primarily as barriers to excavation, leading to their rapid removal, and, it would seem, deliberate concealment of their probable date from their Arab workforce. We now know that Petrie and his field director Starkey collected numerous ‘specimens’ from these types of contexts for craniometric studies. It is hard not to conclude that their interest in these remains lay purely as a source of discrete skulls for comparative anthropometric data and statistical analysis, for which context was seen as irrelevant. The ethics of this behaviour does not seem to have been questioned, either by Petrie or his staff.

Overall Petrie’s work with human remains was very much embedded in, and influenced by, its British colonial context, which determined what was considered ‘archaeology’ and worthy of scientific attention and preservation for the regions under British Mandate control and, by omission, what could be neglected. It is worth noting here that at the time of Petrie’s excavations, discrimination based on the dating of human remains was written into the Antiquities Ordinances of the British Mandate authorities in Palestine. While initial versions of this document did not specifically discuss the treatment of human remains, from 1929, Antiquities Ordinance regulation 2.1.b included as part of the definition of the term ‘antiquity’ any ‘human and animal remains of a date earlier than the year 600 A.D.’ ( Government of Palestine, 1934: 1). This effectively removed any requirement to properly document any burials dating from the Islamic era onwards. Local communities, conversely, were forbidden to establish cemeteries on historical sites without permission from the Department of Antiquities ( Government of Palestine, 1934: 6, Ordinance regulation 18e). These ordinances did not make specific provision for the treatment of pre-existing post-medieval cemeteries on historical sites that fell under excavation, leaving archaeologists free to operate with very loose definitions of the dating and status of graves they encountered in the course of their work. The limited evidence available suggests that potentially modern human remains were being collected from the region without the full awareness or consent of their local communities and probable descendants, a troubling fact that needs to be addressed in the future management and use of these collections.

While the colonial history of archaeology has been acknowledged for decades, many institutional and structural legacies from that period are still in the process of being broken down. In 2018, UCL commissioned an enquiry into its role in the eugenics movement which led to a rebranding of a number of buildings on its campus, including the Pearson Building on Gower Street, which was stripped of its former name ( Janssen, 2020; see also University College London, 2020). There is now ongoing debate as to whether the Petrie Museum of Egyptian Archaeology should similarly change its name, in a move towards greater inclusivity ( Cain, 2024). This is related to wider discussions of ownership and management of cultural heritage and museum collections as a whole, including the stewardship of human remains. The Smithsonian, the curator of one of the largest collections of human remains, adopted an Institution-wide Shared Stewardship and Ethical Returns policy in April 2022, which facilitates the return of collections based on individual circumstances and ethical considerations ( Smithsonian Ethical Returns Working Group, 2022). Similarly, the current staff of the Duckworth Laboratory is doing its part to raise the ethical standards around curating and handling of human remains ( Duckworth Laboratory, 2022).

There has also been discussion of the ethics of continued excavation of human remains and a push to prioritise pre-existing legacy and orphaned collections ( Gregoricka, 2023). Re-examinations of old collections assembled by Petrie and his contemporaries has proved very rewarding, bringing further insights into the health, occupations and cultural practices of ancient Egyptian and Near Eastern populations, not to mention ancestral assessments detached from race-based connotations (e.g., Friedman, 2017; Irish, 2006; Maaranen *et al*, 2022; Miller, 2008; Raxter *et al.*, 2008; Ullinger *et al.*, 2005; Zakrzewski, 2003; Zakrzewski, 2007). Analysis on pre-existing collections, however, should also take place with the consent of descendant communities and rightsholders, but, as Gregoricka (2023) points out, identifying the relevant groups can be difficult; human mobility, lack of records and sifting borders can all interfere with these efforts. Furthermore, different countries and groups, even within the same region, can have vastly different perspectives on the treatment of human remains, not to mention different political agendas.

Sensitivity and cultural awareness are imperative in any work involving human remains, particularly evident with Petrie’s Palestine collections; our investigation of the remains and archives revealed information about these individuals that had become lost, and in some cases even omitted at the time of excavation. Particularly for these individuals, under the current circumstances, it is challenging to suggest the best ethical course of action in terms of analysis and repatriation. While waiting for further decisions, our interaction with the laboratory has provided us with confidence that these matters are taken seriously and with great awareness of cultural and ethnic issues, feeding into the current and future curation of these collections.

## Ethics and consent

This research has been conducted according to the ethical guidelines provided by the British Association of Biological Anthropology and Osteoarchaeology (2019) and the Advisory Panel on the Archaeology of Burials in England (2023), in which human remains are treated with the respect and care they inherently deserve. Consent is not required for this study.

## Data Availability

Zenodo: Bodies of Evidence - Supplementary material, https://doi.org/10.5281/zenodo.14582227 ( Maaranen, 2024). This project contains all osteological data from the recorded human remains, including R code used to analyse the data (referred in text as Supplementary Material. Six files have been provided; fara-ajjul.csv, region.csv, R-fara-ajjul-freqs.R, region.R, Supplemetary Table 1 Tell Fara and Supplementary Table 2 Tell el-Ajjul which contain all data used in the biological distance analysis of this manuscript. Though Nina Maaranen has been named the primary creator of these files, Rachael Sparks has been named as ‘Contributor’ due to her work with contextual information. The data does not contain information about contemporary (living) people; the sample is over 3000 years old. **Supplementary Table 1: Human remains in the Duckworth Collection from identified contexts at Tell Fara.** Sex and age estimations in this table were made by Nina Maaranen, and are based on the skull alone. All sex estimations are tentative and individuals have been divided broadly into adult and subadult. Individuals with sufficient preservation for biodistance analysis have been marked with an asterix, and are discussed further in Section 7.3.3. **Supplementary Table 2: Human remains in the Duckworth Collection from identified contexts at Tell el-ʿAjjul.** Sex and age estimations were made by Nina Maaranen, and are based on the skull alone. All sex estimations are tentative, with broad divisions into adult and subadult categories. Individuals with sufficient preservation for biodistance analysis have been marked with an asterix, and are discussed further in Section 7.3.3. The data and code have been released under the terms of the Creative Commons Attribution 4.0 International license (CC-BY 4.0).

## Footnotes

^1^ There was a single Middle Bronze Age deposit on the edge of the cemetery area containing two vessels, but no skeletal remains (‘Tomb’ 1571). It is not certain that this was a tomb.

^2^ Tomb 1403 contained a brick sarcophagus, but no finds or skeletal remains. Tufnell thought this might have been intended for a Late Bronze II burial ( 1962: 4).

^3^ The ‘600’ numbers have been used throughout this article, in keeping with the field documentation.

^4^ In 1901, Weldon and Pearson founded the journal *Biometrika* ( Challis, 2016: 3), with Galton (1901) writing a paper for the first issue. *Biometrika* became the main publication outlet for many of the studies coming out of Pearson’s lab from then on.

^5^ Only one item appears to be later than this, As.66.4.45 from Tomb 456, which was excavated in the 1933/34 field season at ʿAjjul.

^6^ It had been suggested that Petrie sent skeletal remains directly to the University of Cambridge ( Lovell, 2014: 95), or that Karl Pearson might have organised its transfer himself in the 1940s ( Cockitt, 2014: 14), a logistical impossibility since Pearson died in 1936.

^7^ The exceptions were AS.66.4.8 (P8) and AS.66.4.15 (P15) from Tell Fara, identified as children, and As.64.4.29 (P29) from ʿAjjul, identified as a ‘non-adult’ in Duckworth laboratory notes.

^8^ The stored osteological material contained sheets filled out by Duckworth staff to record sex and age estimations. 85% of these estimations matched those recorded by Maaranen, with disagreements on the sexing of individuals As.66.4.2, As.66.4.11 and As.66.4.14; and on the classification of AS.66.4.4, As.66.4.16 and As.66.4.34 (sexed by the Duckworth, considered indeterminate by Maaranen). Generally, Maaranen’s identifications have erred on the side of caution.
