# Supplementary material for: Bodies of evidence: The human remains from Flinders Petrie’s excavations in British Mandate Palestine
Source: Open Res Eur. 2025 Jan 23;5:22. [Version 1] doi: 10.12688/openreseurope.18758.1 (PMC11871434; doi:10.12688/openreseurope.18758.1)
Supplement: Peer review history

# Reviewer report: Reviewer response for version 1

**DOI:** 10.21956/openreseurope.20293.r50297

**Published:** 2025-02-28

**Authors:** Maria Giovanna Belcastro

**Affiliations:** 1 Alma Mater Studiorum University of Bologna, Bologna, Italy

I have read the manuscript «Bodies of evidence: The human remains from Flinders Petrie’s excavations in British Mandate Palestine» and I appreciated it a lot. It combines the narrative of the excavation and the history of the management and use of the human remains from those Palestinian sites of the archaeologist Flinders Petrie and his contemporary scholars and the most recent anthropological analyses to describe the human variability. I particularly enjoyed reading the paper. The Authors had precisely detailed the historical landscape of the crucial and dramatic period of the onset of the Anthropology as academic discipline in Europe between the late 19th century and the early 20th century, highlighting the ideological perspectives that based Petrie’s work regarding the use of the skulls with phrenological and biometrical purposes to describe and manage the human variability. The human skeletal collections, largely amassed in that period, still brings this heavy legacy that strongly affect the current management. The Authors have shown the relevance to reassess the analyses with the current anthropological methods in the attempt to provide a biological profile to those populations and their relationships throughout the analysis of biodistances. This re-evaluation represents a way to overtake old and dangerous scientific paradigm showing is the same science that adopt new view and perspectives for understanding the human variability, giving back to modern Palestinians a piece of their history particularly important now. This is part of a correct ethical approach of the science.

If any, are all the source data and materials underlying the results available?

Yes

Is the work original in terms of material and argument?

Yes

Is the work clearly and cogently presented?

Yes

Is the argument persuasive and supported by evidence?

Yes

Does the research article contribute to the cultural, historical, social understanding of the field?

Yes

Does it sufficiently engage with relevant methodologies and secondary literature on the topic?

Yes

Reviewer Expertise:

Bioarcheology; biological anthropology; museum anthropological collections;

I confirm that I have read this submission and believe that I have an appropriate level of expertise to confirm that it is of an acceptable scientific standard.

# Reviewer report: Reviewer response for version 1

**DOI:** 10.21956/openreseurope.20293.r50291

**Published:** 2025-02-28

**Authors:** Ariel Gruenthal-Rankin

**Affiliations:** 1 University of Hawaiʻi- West Oʻahu, Kapolei, USA

I commend the authors on this thoroughly researched and important piece related to the history of Flinders Petrie’s work in Palestine and methods of accumulation and curation of bioarchaeological assemblages within museums. I particularly appreciate the deep contextualization of the biological data presented within the paper and the nuance related to concepts like social race, gender expression through contextual artifacts versus sex, and the role that positionality and historical context play in the curation of collections.

 The most notable suggestion for this paper is that it may better serve the reader to be split into at least two papers. One which details the archival research and historical accounts of the excavations of these ‘collections,’ and one related more directly to the biological data collection and analysis of the present study. As it stands, ethically important details which future researchers should consider before working with a collection like this are somewhat obscured by the detail of the historical accounts of the excavations. Excavations should be referenced briefly, as they also affect the ethics of these issues; however, this may be done more effectively by citing a separate paper on these excavations.

 Within the results section, a brief table of descriptive statistics for the current study would be helpful. Although these data are available in supplemental form, the reader would benefit from a summary within text. Because the historical background is so extensive, the biological profile data in the new study portion of this manuscript is overshadowed. Splitting the manuscript into two, as well as expansion of the analysis and discussion sections related to biodistance and sex data would be helpful.

 Within the discussion, at the end of the first paragraph (pg. 27), it would be helpful to engage more deeply with authors who have discussed the “intellectual climate and background” related to museum collection formation, including the work of Jennifer Muller, Rachel Watkins, Aja Lans, and Carlina de la Cova. 

 Along the lines of ethical practices, the authors are applauded for discussing future stewardship of museum collections involving human bodies. It is mentioned (pg. 28, second column, end of first full paragraph) that the Duckworth Laboratory is “…doing its part to raise the ethical standards around curating and handling of human remains.” In light of the unethical way people were incorporated into the collections Petrie established, more detail on the new ethical standards for the Duckworth Laboratory would be appreciated.

If any, are all the source data and materials underlying the results available?

Yes

Is the work original in terms of material and argument?

Yes

Is the work clearly and cogently presented?

Partly

Is the argument persuasive and supported by evidence?

Yes

Does the research article contribute to the cultural, historical, social understanding of the field?

Yes

Does it sufficiently engage with relevant methodologies and secondary literature on the topic?

Partly

Reviewer Expertise:

Bioarchaeology, ethics of skeletal assemblages in museums and teaching institutions.

I confirm that I have read this submission and believe that I have an appropriate level of expertise to confirm that it is of an acceptable scientific standard, however I have significant reservations, as outlined above.

# Reviewer report: Reviewer response for version 1

**DOI:** 10.21956/openreseurope.20293.r50381

**Published:** 2025-02-10

**Authors:** Francisco Curate

**Affiliations:** 1 University of Coimbra, Coimbra, Portugal

It was a pleasure to read the manuscript titled «Bodies of evidence: The human remains from Flinders Petrie’s excavations in British Mandate Palestine», as it offers a detailed narrative of the excavation, under the auspices of the archaeologist William Matthew Flinders Petrie, and post-excavation fate of a sample (or rather, a deeply partitioned set of human bones from different archeological provenances) of human skeletal remains from historical  Palestinian sites. The text is particularly relevant because it draws heavily on the ideological perspectives that based Petrie’s work regarding craniometry or eugenics, and that ultimately had a profound effect on the recovery and curation of the skeletal remains. This profound and serious investigation shows, among other things, that the recovery, study and curation of these human remains was (and still is) contingent to individual and institutional scientific purposes, political standpoints, and, of course, empirical and theoretical knowledge regarding human osteology or the basic bioanthropological methodology kit.  The analysis of biodistance to understand the connection of the more recent skeletal remains (16 ^th^ century [apparently], to late 19 ^th^ century – early 20 ^th^ century) with populations and individuals in the contemporary South Levant is also of anthropological, historical and political relevance, as it could have shown long familial ties.

 I would ask the authors to make some minor adjustments to the text. For example, please reconsider the use of the term “material” when referring to the skeletal remains of deceased individuals. The method for adult age estimation – based on suture obliteration – is ill-described (also, it was not originally published by Buikstra and Ubelaker) and should be more critically addressed as it shows a lack of reliability for age estimation that renders it almost useless in current bioarcheological practice. I would also appreciate a more critical reflection upon the assessment of population affinities, as it a politically contentious procedure, laden with methodological difficulties, questioned by some researchers for its scientific validity.

If any, are all the source data and materials underlying the results available?

Partly

Is the work original in terms of material and argument?

Yes

Is the work clearly and cogently presented?

Yes

Is the argument persuasive and supported by evidence?

Yes

Does the research article contribute to the cultural, historical, social understanding of the field?

Yes

Does it sufficiently engage with relevant methodologies and secondary literature on the topic?

Yes

Reviewer Expertise:

biological anthropology; bioarcheology; forensic anthropology; paleopathology

I confirm that I have read this submission and believe that I have an appropriate level of expertise to confirm that it is of an acceptable scientific standard.
